# Investigation of milk microbiota of healthy and mastitic Sahiwal cattle

**DOI:** 10.1186/s12866-023-03051-0

**Published:** 2023-10-24

**Authors:** Mian Muhammad Salman, Muhammad Nawaz, Tahir Yaqub, Muhammad Hassan Mushtaq

**Affiliations:** 1 Institute of Microbiology, University of Veterinary and Animal Sciences, Lahore, 54000 Pakistan; 2 Department of Epidemiology and Public Health, University of Veterinary and Animal Sciences, Lahore, 54000 Pakistan

**Keywords:** Sahiwal cattle, Milk microbiota, 16S rRNA, Metagenomics, *Firmicutes*, *Proteobacteria*

## Abstract

**Background:**

Sahiwal cattle is an indigenous cattle breed of Pakistan and mastitis is one of the major problems faced by Sahiwal cattle which hinders its production potential. The study was designed to investigate the milk microbiota of healthy and mastitic Sahiwal cattle as part of a multistep project to develop probiotics for the mitigation and control of mastitis. Milk samples of Sahiwal cattle (healthy clinical mastitis and subclinical mastitis) reared under similar husbandry and management practices were processed for 16S rRNA gene base metagenomics analysis.

**Results:**

Results revealed that *Proteobacteria* were dominant in the healthy group and subclinical mastitis group (56.48% and 48.77%, respectively) as compared to the clinical mastitis group (2.68%). In contrast, *Firmicutes* were abundant in the clinical mastitis group (64%) as compared to the healthy and subclinical mastitis groups (15.87% and 38.98%, respectively). Dominant species assigned in the healthy group were *Ignavibacterium album*, *Novosphingobium capsulatum*, *Akkermansia muciniphila* and *Lactobacillus fermentum*.The clinical mastitis group was dominated by *Streptococcus dysgalactiae* and *Corynebacterium bovis*, while subclinical mastitis group included Lactobacillus fermentum and uncultured *acidobacteriales* and *Akkermansia muciniphila* as dominant species. Alpha diversity indices showed higher microbial diversity in the healthy group compared to the clinical and sub-clinical mastitis groups.

**Conclusion:**

It is concluded that the milk microbiota of healthy sahiwal cattle has higher diversity and dominant taxa in the different groups may be used as signature microbes for mastitis susceptibility. *Akkermansia muciniphila* is one of candidate specie that was identified and may be used for development of probiotics.

**Supplementary Information:**

The online version contains supplementary material available at 10.1186/s12866-023-03051-0.

## Background

Sahiwal cattle is an important breed in Pakistan that has the ability to withstand harsh environments and is relatively resistant to diseases [1]. The Food and Agriculture Organization has recommended further development and conservation of Sahiwal cattle [2]. Although this breed is well adapted to tropical countries, its production potential has not been fully explored [1]. Mastitis, inflammation of the udder, is in one of the major causes of decreased milk production and huge economic losses to dairy farmers [3]. Bacteria, one of the major etiology of mastitis, invade the teat canal or mammary gland, multiply and produce toxins which lead to damage to tissues involved in milk secretion [4]. The bacterial agents responsible for causing mastitis are of two types i.e. contagious and environmental. The *Staphylococcus aureus, Streptococcus agalactiae* and *Corynebacterium bovis* are contagious mastitogens whereas Coliforms (*E. coli, Enterobacter and Klebsiella*), *Streptococcus uberis, Streptococcus bovis* and *Streptococcus dysgalactiae* are environmental mastitogens [5]. Mastitis is generally treated with antimicrobial dips and intra-mammary or parenteral antibiotics which not only increase the management cost, it can also further decrease the milk production [5–8] Overuse and misuse of antibiotics also compound the issue of emergence of antibiotic resistant bacterial strains which may also pose a threat to public safety [9]. Advanced culture independent techniques i.e. metagenomics have revealed that internal mammary gland, once considered as a sterile tissue, is habitat to a wide range of opportunistic and commensal bacteria in addition to commonly known mastitogens [10, 11]. Udder microbiome of animals is generally dependent on the environment, animal health status, udder health status and animal breed [11, 12]. Gut microbiota of animal may also migrate through the entero-mammary pathway and contribute to the udder microbiome [13]. The non aureus Staphylococci (NAS), commensal species, which are frequently detected in cattle milk, are paradoxical in maintaining udder homeostasis [14–16]. Some NAS (*Staph. chromogenes*) provide protection against mastitogens by producing bacteriocins [17]. The role of other non mastitogenic species and beneficial microbes of gut microbiota is not fully understood yet, but it has been suggested recently that microbial dysbiosis in udder plays an important role in development of mastitis [18], therefore maintaining a healthy microbiome of udder and internal mammary gland may reduce and control the mastitogens.

There are only few previous reports which have analyzed and compared the milk or udder microbiome of healthy and mastitic cattle [12, 19, 20]. Identification of signature bacterial taxa for healthy, subclinical and clinical mastitic cattle may provide an option of augmentation of udder microbiota with signature microbial species as probiotics to ameliorate the udder dysbiosis and control the mastitis. To the best of the author’s knowledge, the udder or milk microbiome of Sahiwal cattle breed has not been studied. Therefore, the present study was designed to explore the milk microbiota of healthy and mastitic Sahiwal cattle by metagenomics, in an effort to identify signature bacterial taxa, as a first step in a multi-step project, to develop probiotics for the mitigation of bovine mastitis.

## Methods

### Selection of households and animals

Sampling for this study was conducted in the Okara district of Punjab, which has a high population of Sahiwal cattle. Four households with lactating animals that were managed under similar husbandry and management practices were selected (Supplementary table S1). Animals between 3–6 years of age, with at least one lactation and no history of antibiotic treatment within the last 15 days, were included in the study. Milk samples were collected from all animals and screened for mastitis. A total of 15 samples, comprising 5 samples each from healthy, clinical mastitis, and subclinical mastitis groups, were selected randomly for metagenomic analysis (Supplementary table S2).

### Sample collection and processing

Information related to animals and households was collected prior to sample collection on a predesigned performa. The udder of each animal was inspected for presence of any inflammation while milk samples were observed physically for the presence of flakes, blood, watery secretion, viscosity and appearance. Animals with inflammatory signs in the udder or abnormality in milk were classified as having clinical mastitis. Rectal Temperature of animal was checked to rule out any systemic involvement. Following physical examination the first two streaks of milk were discarded from each teat and milk sample was tested using California mastitis test. Based on physical examination of animals, its udder, California mastitis test (CMT) and somatic cell counts milk sample was grouped into healthy and subclinical mastitis. Subclinical status was allotted based on absence of any inflammatory signs in udder and any abnormality in milk but were positive on CMT and having more than 2,00,000 somatic cell count while animals with no inflammation, no abnormality in milk, negative on CMT and less than 2,00,000 somatic cell count were classified as healthy. After confirming the udder health status of the animal, the udder was cleaned any dirt was removed and washed with water, dried with a towel and swabbed with 70% ethanol. Then 15 ml milk sample was collected in falcon tube following the protocol described in the laboratory and field handbook on bovine mastitis [21]. The Milk samples were transported to Probiotics Research Laboratory, Institute of Microbiology, UVAS Lahore at 4ºC and were stored at -80ºC prior to further processing.

### DNA extraction

All the steps for DNA extraction from milk samples were carried out in the same biosafety cabinet in the same laboratory using the same procedure under similar conditions. The samples were brought to room temperature prior processing it for DNA extraction. For extraction DNA from milk, pellet method adopted by Yap et al. [22] was used.15 ml milk sample was centrifuged at 4500 × g for 20 min at 4 °C.cream and supernatant was removed carefully and discarded. After this step the samples were washed two times by adding sterile phosphate-buffered saline (PBS) and centrifuging it at speed of 13,000 × g for 1 min. The pellet was the used for DNA extraction after careful removal of supernatant. All the DNA extraction of milk samples and negative control (Phosphate buffer saline) was carried out using DNeasy PowerSoil Kit (Qiagen) following manufacturer recommendations with slight modifications. The concentration and limpidity of the extracted DNA was checked at 260/280 nm using Multiskan sky microplate spectrophotometer (Thermoscientific) and samples having O.D between 1.6–1.9 were selected for 16S r RNA gene base metagenomic analysis.

### DNA quality control and sequence library preparation

The DNA of each sample was properly labeled and packed and sent for 16S rRNA gene base metagenomic sequencing to (Macrogen, Seoul, South Korea). The quantity check for double stranded DNA was performed using DNA binding dye (Invitrogen, cat.#P7589) (Walthan, United States) through vector 3 fluorometry (Waltham, United States).The amplicon were generated using standard protocols using specific primers (Forward primer TCGTCGGCAGCGTCAGATGTGTATAAGAGACAGCCTACGGGNGGCWGCAG, Reverse Primer GTCTCGTGGGCTCGGAGATGTGTATAAGAGACAGGACTACHVGGGTATCTAATCC targeting V3 and V4 region of 16S rRNA gene [23]. Illumina DNA prep (Illumina, San Diego, United states) was used for 2X300 bp Miseq library preparation following the procedure adopted in illumina 16S Metagenomic Sequencing Library Preparation Part # 15044223 Rev. B protocol.

### Bioinformatics analysis

16 s rRNA gene sequences that were received were in demultiplexed, pair end read form. They were then subject to analysis using analytical bioinformatics pipeline QIIME 2 (Quantitative insight into Microbial Ecology) version 2.2020.6 software package [24]. Pair end reads FASTA, files were imported using the manifest file import method described for pair end demultiplexed data in QIIME 2 guidelines. Amongst the available donizing methods in QIIME 2 for filtering of the noisy data q-dada2 was used [25]. Trimming criteria of 300 bp was selected and reads which were longer than this length along with chimeras were subjected to exclusion. The frequencies of OTU (operational taxonomic unit) and its corresponding representative tables were created by using the QIIME tool. The taxonomy assignment of the OTU sequences was carried out using a q-2 feature classifier i.e. classify-sklearn which is a machine learning based classification method available in QIIME 2. Silva data base (Silva 138) was used to train the classifier using a threshold identity of 99%. Visualization of data of each sample was done using taxa bar plots for different levels.

### Statistical analysis

The data obtained for each level in the form of OTU numbers for each sample at different levels were converted into percentage abundance in Microsoft Excel (Microsoft Corp., Redmond, WA). The difference in percentage abundance data in major taxa at every level was analyzed using one ANOVA (Analysis of variance) and graphs were prepared using Graph pad Prism (GraphPad Software, USA) software and Microsoft Excel. Shannon diversity index (H) and Simpson’s index (D) which are based on species richness and evenness and richness base diversity indices such as observed features, and Chao 1 were used for the estimation of alpha diversity. Nonparametric Kruskal–Wallis test was used to compare alpha-diversity indices. Beta diversity indices including quantitative non phylogenetic diversity metrics (Jaccard distance, Bray–Curtis) and phylogenetic beta diversity metrics (weighted and unweighted unifrac)were used to calculate beta diversity. The beta diversity indices were visualized using Principal component analysis graph for observing the relationship based on beta diversity indices. Permutational ANOVA (PERMANOVA) test was used for analyzing beta diversity metrics.

## Results

### Taxonomic profile at phylum level

The milk microbiome profile of Sahiwal cattle analysis revealed a total of 22 phyla, 49 classes, 132 orders, 207 families, 309 genera and 168 species with different levels of distribution among the groups. All the 15 samples comprised of 421,400 clean reads having a range of 11,022- 43,988 reads (median reads = 25,602 per sample) distributed among all groups. The OTU found in healthy group were 1,11,811, in clinical mastitis group 1,80,126 and 1,29, 463 in sub clinical mastitis group.

**Fig. 1:**
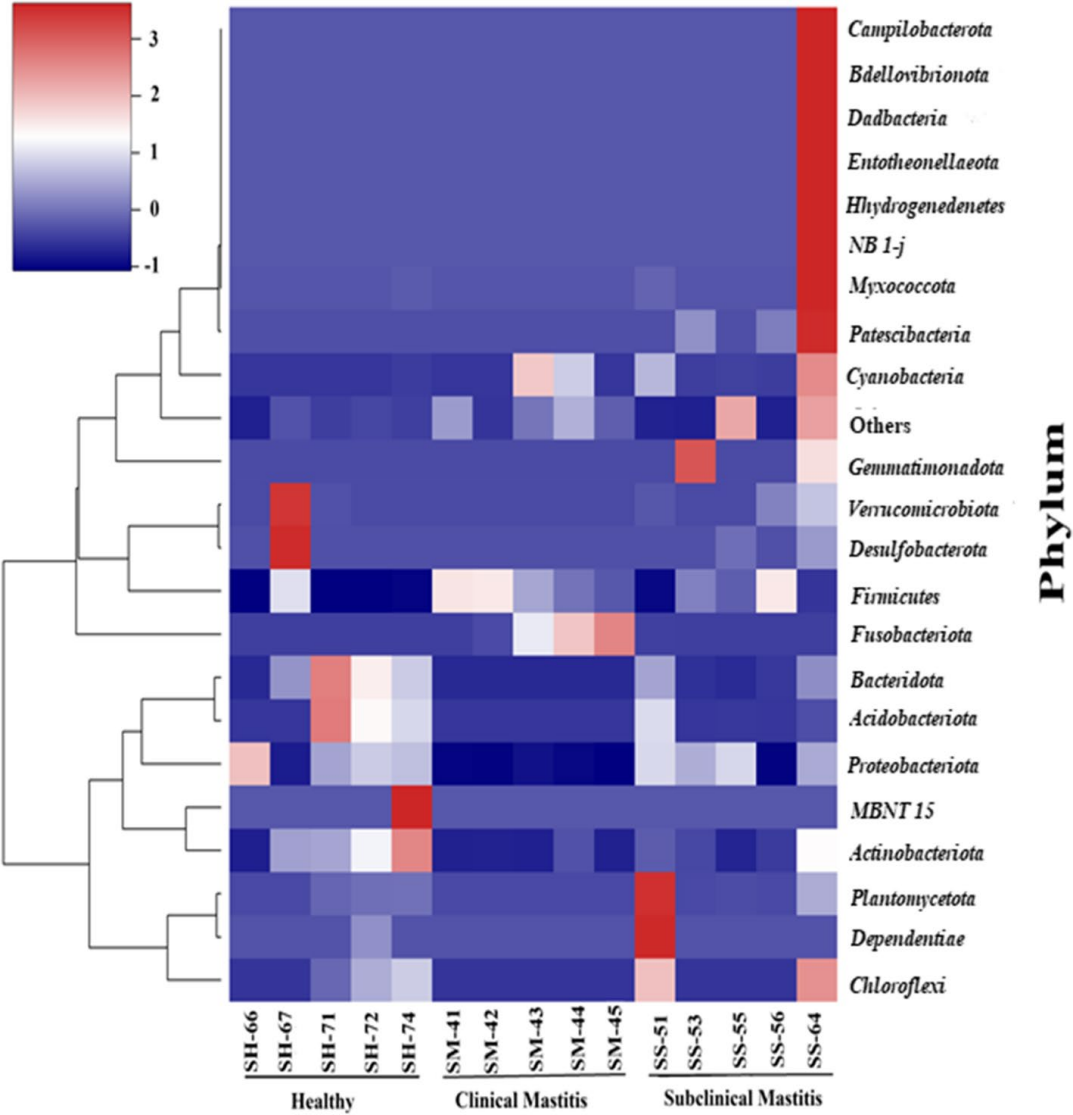
Heat-map plot of the relative abundance of different phyla in Sahiwal cattle Milk Microbiota. Healthy (*n* = 5), Clinical Mastitis (*n* = 5) and Subclinical Mastitis (*n* = 5). Dendrograms show the clustering of different phyla in the same group

**Fig. 2:**
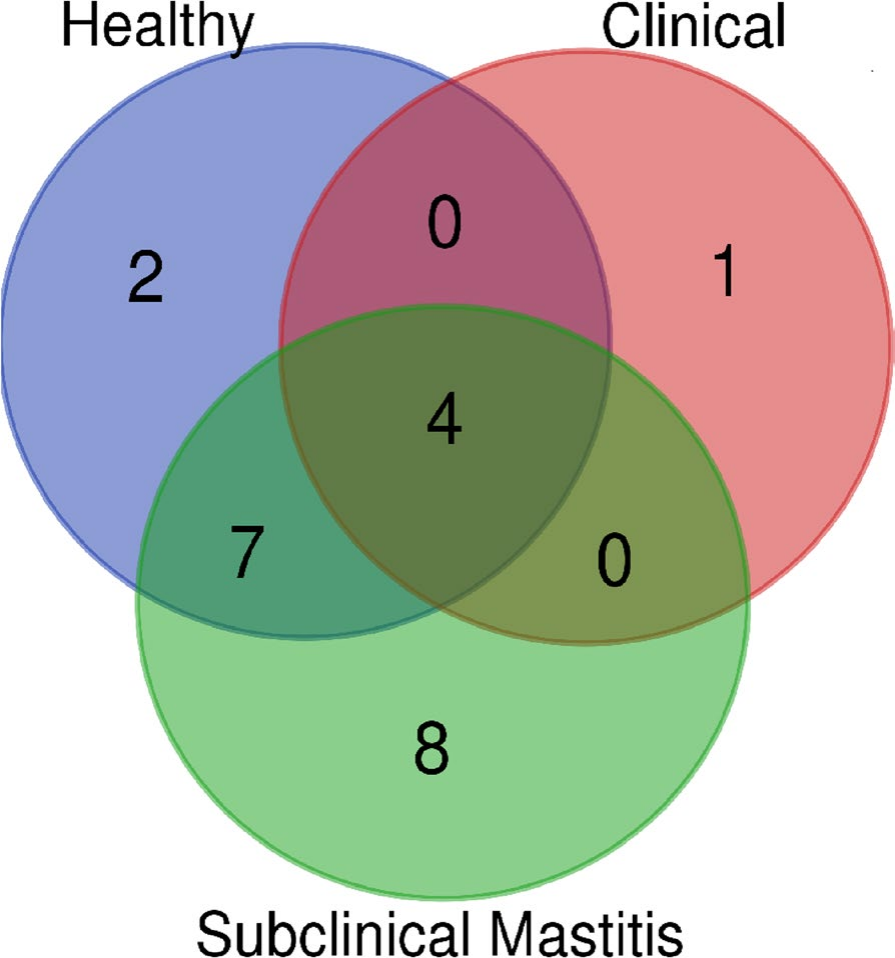
Venn diagram showing distinct and shared phyla among different groups in milk samples of Sahiwal cattle. Healthy (*n* = 5), Clinical Mastitis (*n* = 5) and Subclinical Mastitis (*n* = 5)

**Fig. 3:**
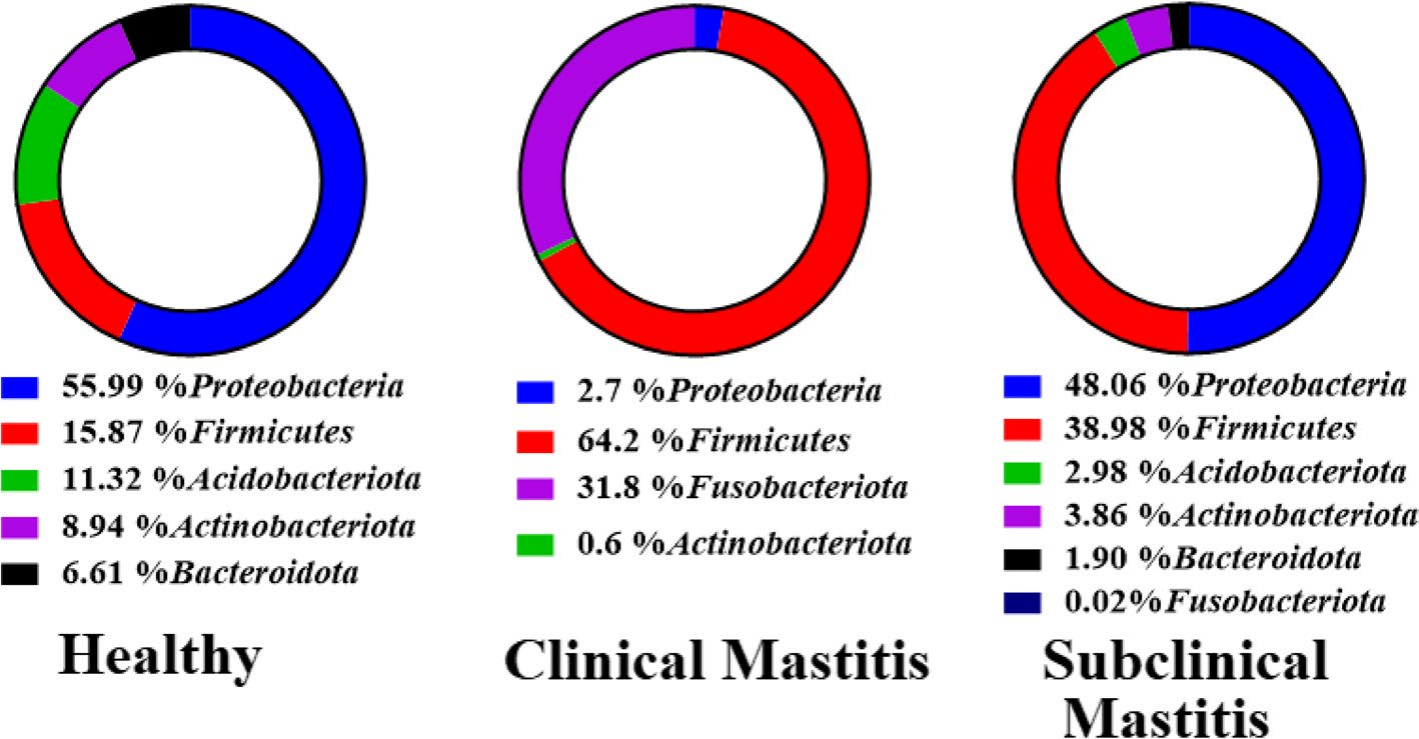
Donut graph of Percentage abundance base representation of taxonomic distribution at phylum level of Sahiwal cattle milk. Different colors represent different phyla amongst the three groups

The Phylum level taxonomy assignment of the three groups revealed 22 phyla (Fig. 1). Only 4 phyla were shared among all the groups including *Proteobacteria, Firmicutes, Actinobacteria* and *Cyanobacteria*. The numbers of distinct phyla in subclinical mastitis group were eight, in the healthy group two and clinical mastitis group only one phylum was distinct (Fig. 2). Although the distribution of microbiota amongst different phyla varied but six of them comprised more than 90% of the total phyla with different abundance. The percentage abundance of *Proteobacteria* was highest in the healthy group (56%) followed by a percentage abundance of 48.06% in the subclinical mastitis group and significantly lower (*p* < 0.05) abundance was detected in clinical mastitis group (2.68%) (Table 1). *Firmicutes* dominance was observed in clinical mastitis group (62.24%) and subclinical mastitis group (39.98%), it was detected in lower abundance in healthy group (15.87%). *Acidobacteriota* abundance was observed to be highest in healthy animals (11.32%) followed by (2.98%) in subclinical group while it was not detected in clinical mastitis group. The percentage abundance of *Bacteroidota* was significantly higher (*p* < 0.05) in healthy group (6.61%), it was 1.90% in subclinical mastitis group while in clinical mastitis group it was not detected. The percentage abundance of *Fusobacteriota* was significantly higher (*p* < 0.05) in clinical mastitis group (31.79%) as compared to subclinical mastitis group (0.02%) while it was not detected in healthy group. The percentage abundance of *Actinobacteriota* was 8.94%, 3.86% and 0.61% in healthy, sub clinical mastitis and clinical mastitis groups respectively (Fig. 3) with no significant difference (*p* < 0.05).

**Table 1:** Percent abundance (mean) comparison at phylum taxonomic level in milk samples from Sahiwal cattle grouped as healthy, clinical mastitis and with subclinical mastitis only signifcantly different taxa are denoted with letters

**Taxa**	**Healthy**			**Clinical Mastitis**			**Subclinical Mastitis**		
**Phylum**	**Mean**		**SEM**	**Mean**		**SEM**	**Mean**		**SEM**
*Proteobacteria*	55.99	^a^	14.43	2.68	^b^	0.90	48.06	^a^	11.95
*Firmicutes*	15.87		15.13	64.24		14.01	38.98		15.76
*Acidobacteriota*	11.32		5.25	0.00		0.00	2.98		2.51
*Actinobacteriota*	8.94		3.19	0.61		0.51	3.86		2.14
*Bacteroidota*	6.61	^a^	2.33	0.00	^b^	0.00	1.90	^ab^	0.99
*Fusobacteriota*	0.00	^b^	0.00	31.79	^a^	13.62	0.02	^b^	0.02

*SEM* standard error mean, Different letters (a,b) in the same rows denotes differences between means for *p*-value < 0.05

### Taxonomic profile at order level

**Fig. 4:**
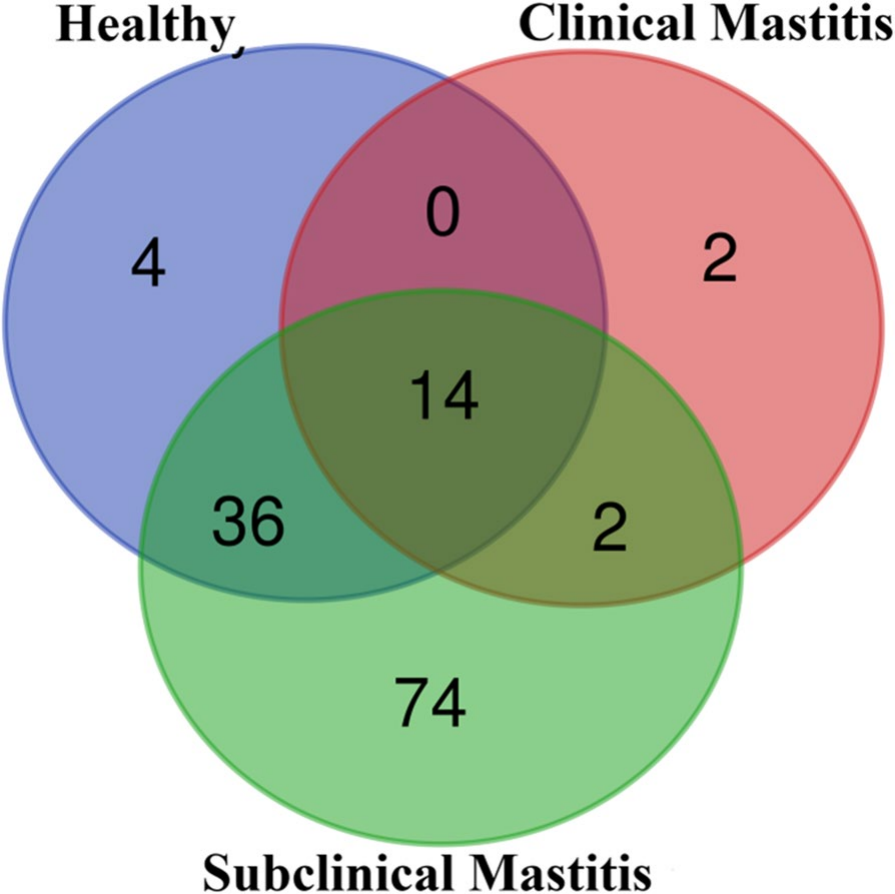
Venn diagram showing distinct and shared phyla among different groups in milk samples of Sahiwal cattle. healthy (*n* = 5), clinical mastitis (*n* = 5) and subclinical mastitis (*n* = 5)

**Fig. 5:**
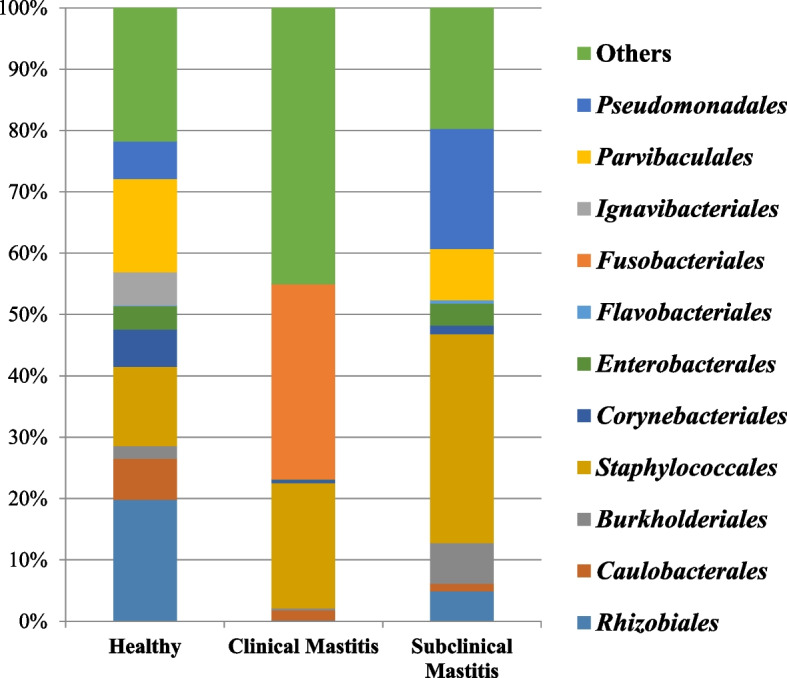
Taxa bar plot representing taxonomic distribution at Order level of Sahiwal cattle milk. Different colors represent different orders amongst the three groups

The orders shared between all the groups were 14 with distinct orders being highest in subclinical mastitis group (Fig. 4). Amongst the orders *Rhizobiales, Parvibaculales* and *Staphylococcales* were the abundant orders identified in the healthy group. *Fusobacteriales* and *Staphylococcales* were most abundant in clinical mastitis group while the subclinical mastitis group was dominated by *Staphylococcales* and *Pseudomonadales* (Table 2 and Supplementary figure S2). *Rhizobiales* being the highest in terms of percentage abundance in the healthy group (19.82%) whereas a low percentage abundance was recorded in healthy group (0.17%) and 4.86% in subclinical mastitis group. The percentage abundance of *Caulobacteriales* showed a non significant difference (*p* < 0.05) between the groups, with most highest in the healthy group (6.65%) followed by clinical mastitis group (1.60%) and the least abundance in subclinical mastitis group (1.27%). Order *Burkholderiales* was dominant in subclinical mastitis group (6.64%) while clinical mastitis group was the lowest in terms of abundance (0.38%), Healthy group showed abundance of 2.11% of *Burkholderiales*. Although percentage abundance of order *Staphylococcales* was non significantly different amongst all the three groups. It was observed to be present in highest abundance in subclinical mastitis group (34.05%) followed by clinical mastitis group (20.38%) and healthy group (12.92%). *Corynebacteriales* showed a non significant difference in percentage abundance in all the groups being the most highest in healthy group (6.06%), in clinical mastitis group having a low abundance of (0.55%). Compared to the clinical mastitis group the percentage abundance of *Corynebacteriales* was high in subclinical mastitis group (1.41%) but it was low as compared to the healthy group. The abundance of order *Enterobacteriales* followed the same trend as found in *Corynebacteriales* but the values were 3.79% for healthy, 0.07% in clinical mastitis group and 3.59% in subclinical mastitis group. *Fusobacteriales* was significantly higher (*p* < 0.05) in clinical mastitis group (31.79%), lower in subclinical mastitis group (0.02%), while it was not detected in healthy group. *Ignavibacteriales* was only detected in healthy group while in other two groups it was not detected. *Parvibaculales* was not detected in clinical mastitis group while in healthy group (15.19%) it was the third most abundant order and the percentage abundance in subclinical mastitis group recorded was 8.30%. Subclinical mastitis group contained *Pseudomonadales* as 2nd most abundant (19.65%) whereas healthy group was found to have a percentage abundance of 6.11% and in clinical mastitis group (0.02%) (Fig. 5).

**Table 2:** Percent abundance (mean) comparison of mean at Order taxonomic level in milk samples from Sahiwal cattle grouped as healthy, clinical mastitis and with subclinical mastitis. Only signifcantly different taxa are denoted with letters

	**Healthy**			**Clinical Mastitis**			**Subclinical Mastitis**		
**Order**	**Mean**		**SEM**	**Mean**		**SEM**	**Mean**		**SEM**
*Rhizobiales*	19.82		11.48	0.17		0.05	4.86		2.20
*Caulobacteriales*	6.65		5.89	1.60		0.76	1.27		0.51
*Burkholderiales*	2.11		0.68	0.38		0.15	6.64		5.17
*Staphylococcales*	12.92		12.73	20.38		18.73	34.05		16.50
*Corynebacteriales*	6.06		2.82	0.55		0.52	1.41		1.10
*Enterobacteriales*	3.79		2.48	0.07		0.02	3.59		2.14
*Flavobacteriales*	0.12		0.05	0.00		0.00	0.52		0.38
*Fusobacteriales*	0.00	b	0.00	31.79	a	13.62	0.02	b	0.02
*Ignavibacteriales*	5.46		2.53	0.00		0.00	0.00		0.00
*Parvibaculales*	15.19		6.28	0.00		0.00	8.30		8.29
*Pseudomonadales*	6.11		2.57	0.02		0.02	19.65		10.61

*SEM* standard error mean, Different letters(a,b) in the same rows denotes differences between means for *p*-value < 0.05

### Taxonomic profile at genus level

The genus level assignment of milk microbiome revealed a total of 309 genera with different abundance levels in all three groups. The abundant genera detected in healthy group included *Parvibaculum, Staphylococcus, Ignavibacterium and Pseudomonas.* Clinical mastitis group was dominated by *Streptococcus, Caviibacter and Staphylococcus*. Subclinical mastitis group included *Staphylococcus, Pseudomonas and Parvibaculum* as the top three abundant genera. *Genus Novosphingobium* was detected in all three groups with a higher percentage abundance detected in healthy group (0.22%) followed by clinical mastitis group (0.13%) and being the least abundant in subclinical mastitis group (0.06%). *Caviibacter* genus showed the significantly highest abundance ((*p* < 0.05) in clinical mastitis group (31.80%) as compared to other genera and it was also the highest as comparedto other groups because it was not detected in other groups*.* The pattern of percentage abundance of *Corynebacterium* genus showed a trend of being highest in subclinical mastitis group (0.79%) followed by healthy group (0.73%) whereas being the lowest in clinical mastitis group (0.55%). *Escherichia-Shigella* genus was observed as the least abundant in clinical mastitis group (0.49%) while subclinical group (0.07%) showed a high abundance as compared to clinical mastitis group while healthy group (1.16%) appeared as the most abundant group in terms of *Escherichia-Shigella*. *Ignavibacterium genus was* only detected in healthy group (5.7%) and was significantly higher as compared to other groups*. *The percentage abundance of *Lactobacillus* was in decreasing order from clinical mastitis group, subclinical mastitis group and healthy group. *Parvibaculum* genus was identified as the most abundant genus in healthy group, in subclinical mastitis group it occurred as the 3^rd^ abundant genus detected whereas it was found absent in clinical mastitis group*. Pseudomonas* genus appeared the 2^nd^ most abundant genera in subclinical mastitis group (18.56%) while the percentage abundance of *Pseudomonas* in healthy and clinical mastitis group was 5.35% and 0.01% respectively*. Staphylococcus* ranked 1^st^, 2^nd^ and 3^rd^ amongst different genera in healthy (13.43%), Clinical mastitis (20.39%) and subclinical mastitis group (34.36%) respectively*. Streptococcus* was only detected in clinical mastitis group (42.68%) and was significantly higher (*p* < 0.05) as compared to other groups and ranked as the top genera of the group (Table 3 and Supplemntary figure S4).

**Table 3:** Percent abundance (mean) comparison of mean at Genus taxonomic level in milk samples from Sahiwal cattle grouped as healthy, clinical mastitis and with subclinical mastitis only signifcantly different taxa are denoted with letters

**Taxa**	**Healthy**			**Clinical Mastitis**			**Subclinical Mastitis**		
**Genus**	**Mean**		**SEM**	**Mean**		**SEM**	**Mean**		**SEM**
*Novosphingobium*	0.22		0.08	0.13		0.03	0.06		0.04
*Caviibacter*	0	b	0	31.80	a	13.62	0	b	0
*Corynebacterium*	0.73		0.45	0.55		0.52	0.79		0.66
*Escherichia-Shigella*	1.16		0.93	0.07		0.02	0.49		0.08
*Ignavibacterium*	5.76	a	2.61	0	b	0	0	b	0
*Lactobacillus*	0.34		0.22	0.65		0.38	0.44		0.27
*Parvibaculum*	16.21		6.69	0		0	8.62		8.62
*Pseudomonas*	5.35		2.73	0.01		0.01	18.56		10.36
*Staphylococcus*	13.43		13.36	20.39		18.74	34.36		16.59
*Streptococcus*	0	b	0	42.68	a	15.10	0	b	0

*SEM* standard error mean, Different letters(a,b) in the same rows denotes differences between means for *p*-value < 0.05

### Taxonomic profile at specie level

A total of 168 bacterial species were found in all three groups after taxonomy assignment at the specie level. Uncultivated species of many phyla were given a cornucopia of classification. In some other cases, no taxonomy was assigned, and it was regarded as unknown. In our study, species were not resolved to the same degree as other taxonomic levels. While species percentage abundance varied between the groups, many species were not classified according to any particular taxonomic rank (Supplemntary figure S5). While some assigned species were assigned at higher levels of taxonomy, they lacked specie classification i.e. phylum, class, order and genus. We found that different groups had distinct representations of species from various phyla. The diversity among various groups was greater at the species level, such as *Staphylococcus* was identified at genus level but no specie of *Staphylococcus* was assigned. *Ignavibacterium album* (15.72%) was predominant in the healthy group followed by *Novosphingobium capsulatum *(10.42%), *Akkermansia muciniphila* (3.57%), uncultured *Acidobacteriales *(3.06%), uncultured *Rubrobacteraceae* (2.43%), uncultured *Actinobacterium* (0.64%), uncultured Phyllobacteriaceae (0.49%), Pseudomonas sp (0.16%), *Lactobacillus fermentum* (0.12%) and uncultured *Rhizobiales* (0.11%). *Corynebacterium bovis* and *Streptococcus dysgalactiae* were not detected in healthy group. The clinical mastitis group was dominated by *Streptococcus dysgalactiae *(49.15%) and *Corynebacterium bovis* (1.33%) along with *Novosphingobium capsulatum *(0.73%) while other species found in healthy and subclinical mastitis group were not detected in the group. Subclinical mastitis group was dominated by species that were not detected in clinical mastitis group while the dominant species that were detected in clinical mastitis group was not detected in clinical mastitis group. In subclinical mastitis group *Lactobacillus fermentum* (5.71%)*,* uncultured *Acidobacteriales *(4.95%), *Akkermansia muciniphila *(1.63%)*, Pseudomonas sp *(1.04%) and uncultured *Actinobacterium *(0.32%) were identified. Amongst the healthy group *Ignavibacterium album* was significantly higher (*p* < 0.05) as compared to the clinical mastitis group and subclinical mastitis group. In the clinical mastitis group the dominant specie i.e. *Streptococcus dysgalactiae* was significantly higher (*p* < 0.05) in the group as compared to the healthy group and subclinical mastitis group. The other species that was identified to be significantly higher (*p* < 0.05) in healthy group as compared to the clinical mastitis group was uncultured *Rhizobiales*.

The heat maps plots and group wise percentage tables abundance at Class and Family level is provided as Supplementary figure S 1 and S 4 and Supplementary Tables 10 and 11 respectively. While percentage abundance of individual samples at all levels are provided as Supplementary Table. 4– 9.

### Alpha diversity analysis of Sahiwal cattle milk microbiota

**Fig. 6:**
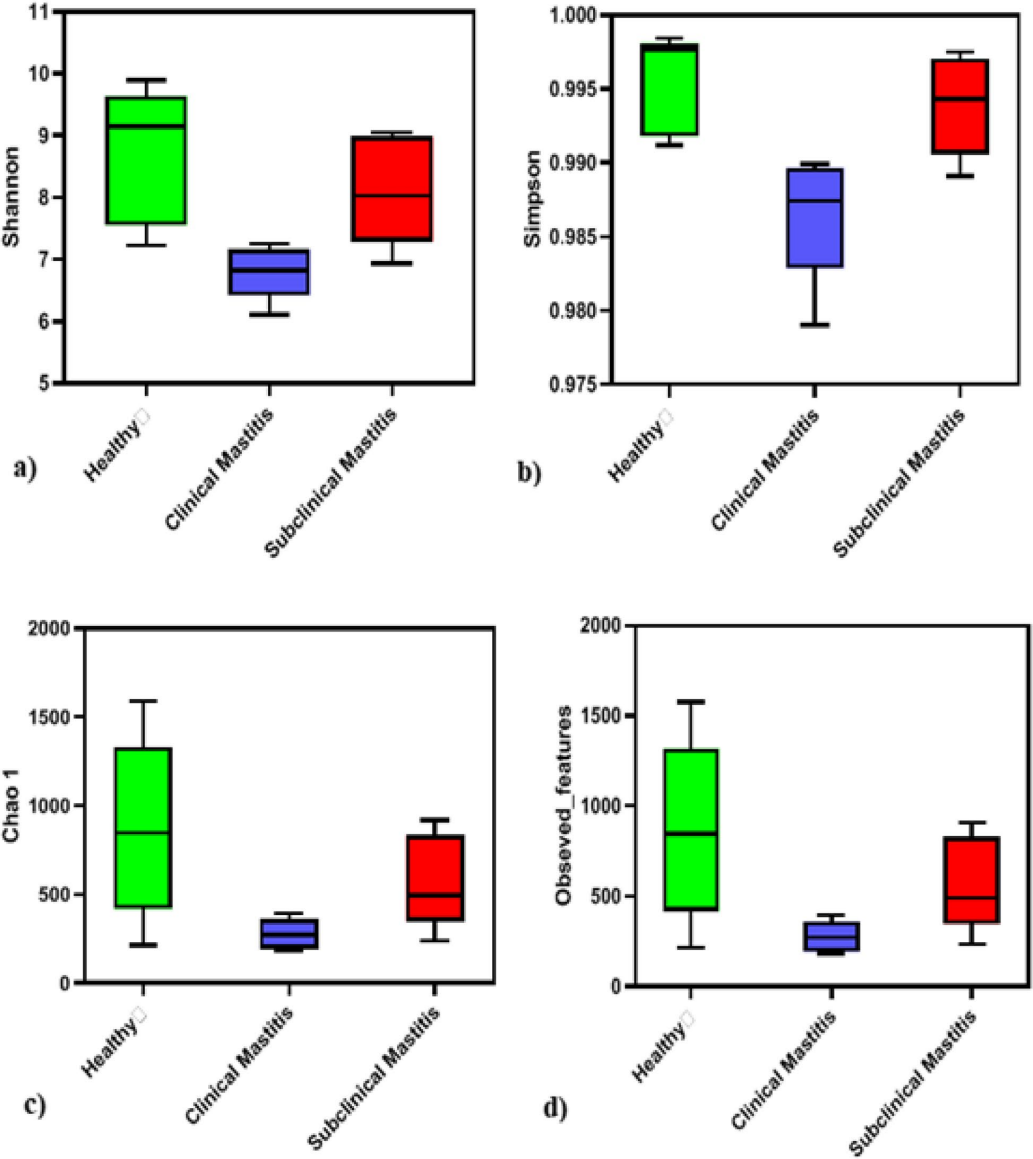
Alpha diversities of the milk microbiome collected from Sahiwal cattle (healthy = green, clinical Mastitis = blue and subclinical Mastitis group = red) calculated by **a**) Shannon index **b**) Simpson **c**) Chao 1 **d**) observed species

Assessment of the three study groups using different alpha diversity metrics depicts a clear picture in terms of the microbial diversity in these groups. Shannon and Simpson diversity indices which deduce additional information about the community composition in addition to just species richness or evenness. This study shows that the microbial diversity was higher in healthy group as compared to clinical mastitis and subclinical mastitis group while lowest diversity was observed in clinical mastitis group. Chao 1 and observed features are diversity indices which are abundance base coverage estimators of specie richness also shows that alpha diversity was highest in healthy group as compared to other groups while being the lowest in clinical mastitis group (Fig. 6).Simpson and Shannon microbial diversity indices were significantly higher in healthy group (Table 4).

**Table 4:** Alpha diversity indices in healthy, clinical and subclinical mastitis in Sahiwal milk samples

Parameter	Healthy	Clinical Mastitis	Subclinical Mastitis	Kruskal Wallis	*P*-value
Shannon mean ± SEM	8.708 ± 0.4964	6.798 ± 0.1952	8.118 ± 0.39	7.98	0.0105*
Simpson mean ± SEM	0.9955 ± 0.0015	0.98648 ± 0.0020	0.99392 ± 0.0015	8.78	0.0050**
Chao1 mean ± SEM	868.57 ± 228.87	277.02 ± 39.28	572.032 ± 118.94	5.82	0.0544
Observed features mean ± SEM	864.4 ± 226.57	277 ± 39.26	568.8 ± 117.80	5.82	0.0544

*p*-values were considered significant at *p* = 0.05 (*), 0.01 (**), 0.001(***)

### Beta diversity analysis of sahiwal milk microbiota

**Fig. 7:**
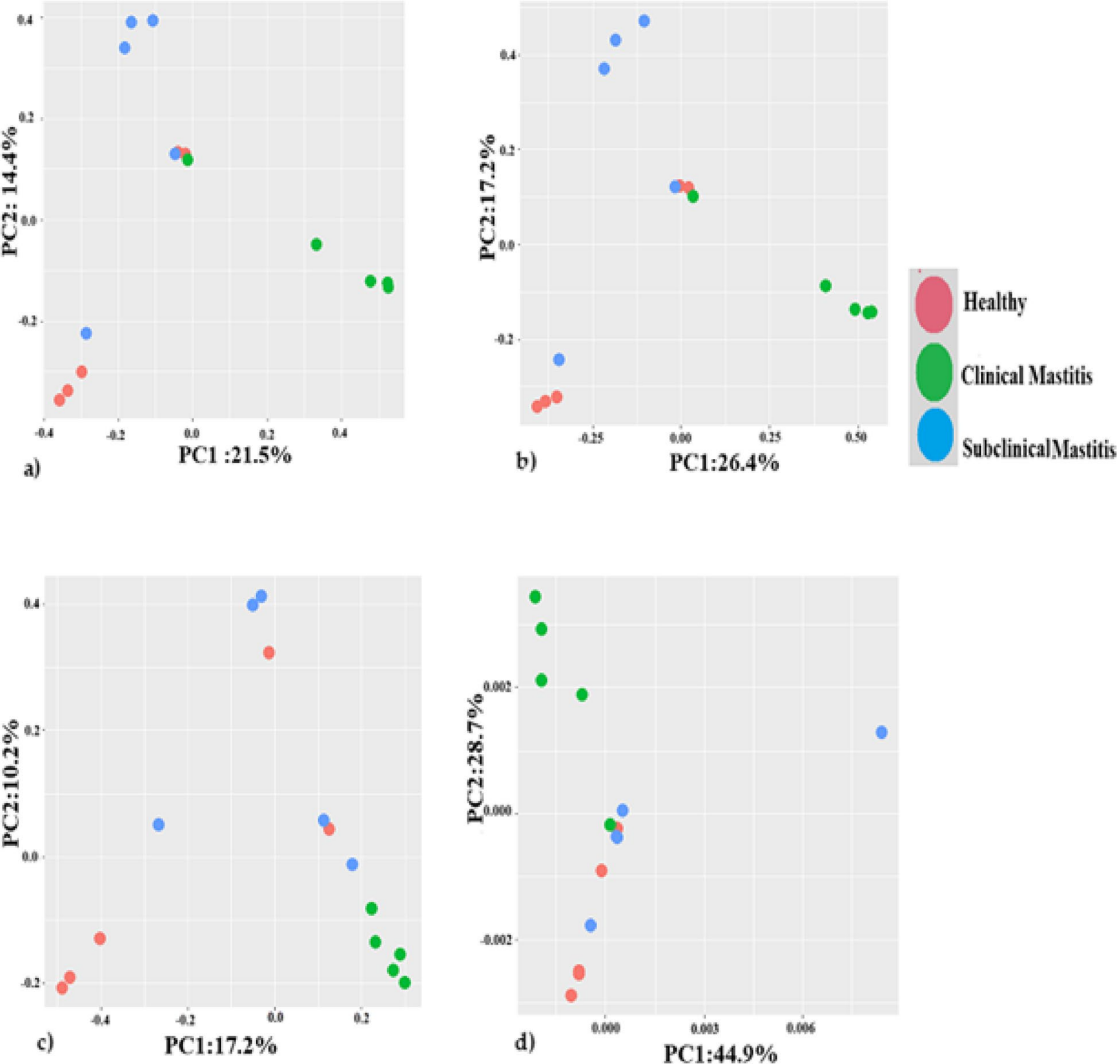
Beta diversity indices for Sahiwal milk of different groups. Principal component analysis plots based on **a**) Jaccard index **b**) Bray Curtis dissimilarity index. **c**)  Unweighted **d**) Weighted unifrac metrics

The beta diversity indices (Jaccard Index and Bray Curtis dissimilarity index) shows a same pattern where in 80% (4 out of 5) healthy samples were clustered together separately from other samples of the two groups. Similarly, 60% (3 out of 5) of subclinical samples were clustered separately. while 60% (3 out of 5) samples of healthy group were clustered with 20% (1 out of 5) of subclinical samples seperatly. 40% (2 out of 5) of healthy, 20% (1 out of 5) clinical and 20% (1 out of 5) subclinical samples were clustered together separately from other samples. Weighted and unweighted unifrac Principal Component analysis plot shows that all clinical mastitis samples are clustered separately from the other two groups while there is clustering of 60% (3 out of 5) healthy samples in unweighted unifrac PCA plot and 40% are clustered together in weighted unifrac PCA plot. While remaining healthy and subclinical mastitis samples have been found to have mixed diversity patterns (Fig. 7).

## Discussion

Bovine mastitis is a multi etiological inflammatory condition of udder resulting from infectious and non infectious agents and it has major contribution in the economic losses to the farmer worldwide [26]. Identification of microorganisms using classical culture base techniques has the disadvantage that only 1% of the microorganisms are culturable [27]. The next generation sequencing tools such 16S rRNA gene base metagenomic techniques can help in exploration of the microbiota associated with mastitis [10], especially in unexplored breeds such as Sahiwal. Thus in turn it will help in defining control strategies for mastitis according to the etiological agent. In this study samples were selected from households having same managemental and husbandry practices so the effect of these variables seems to be very less in defining the microbial composition of this breed. The abundant phyla detected in healthy samples was *Proteobacteria* followed by *Firmicutes* which is an agreement with the findings of Pang et al. [28]. The finding are not consistent with some studies [20, 29]. In our study clinical mastitis group showed a high percent abundance of *Fusobacteriota* and *Fusobacteriales* at phylum and order level respectively in clinical mastitis group. The opportunistic nature of *Fusobacteria* species in mastitis has been explored in different studies and has been identified in milk samples of cows suffering from clinical mastitis [30]. The pattern of occurrence of two major phyla *Proteobacteria* being highest and *Firmicutes* being lowest in subclinical mastitis in this study is in agreement with the findings of the research work [28, 31]. *Caulobacteriales* abundance was recorded in all three groups but its abundance was a bit higher in healthy samples due to its high abundance in one sample (SH-66) the possible reason as suggested by Oikonomou [30] may be due to water contamination of sample or due to their presence in high numbers in milking parlor.

Although *Staphylococcus* species were not identified at specie level but order *Staphylococcales* and *Staphylococcus* genus has been detected and its abundance has been observed to be highest in subclinical mastitis followed by clinical mastitis samples, it has also been detected in healthy animals. The abundance of *Staphylococcus* has been shown to be high in subclinical samples than the healthy animals [29] its adaption and presence in healthy animals in high number has been attributed to their virulence factor such as adhesion, colonization and biofilm production [32]. The occurrence of high percentage of *Staphylococcus* in subclinical mastitis is because of the infection initially begins in form of subclinical mastitis form and then may progress to clinical stage [29] there is also a concept thatall NAS((non aureus *staphylococcus*) species does not cause mastitis some of NAS species provide protection from mastitic pathogens through bacteriocins and they have been detected in cattle milk [33].

*Streptococcus* was identified at genus level and *Streptococcus dysgalactiae* specie has been identified in high abundance in clinical mastitis samples in our study. *Streptococcus* genus include *Streptococcus agalactiae, Streptococcus uberis* and *Streptococcus dysgalactiae*, responsible for causing mastitis [34, 35]. *Streptococcus dysgalactiae* is known to be transmitted by flies [36] and dairy farms play an important role by serving as feeding and breeding place for these flies in the form of manure and animal feces [37] thus our findings suggest that flies have a major role in occurrence of mastitis due to *Streptococcus dysgalactiae* due to high number of flies population being observed at households having Sahiwal cattle.

In our study, we found *Akkermansia muciniphila* in healthy and subclinical milk samples. it has the ability to degrade mucin and make use of carbon and nitrogen [38] its abundance has been reported to be negatively associated with occurrence of obesity, inflammation and diabetes in studies on humans and animals [39].  Studies on mice fed with goat and cattle milk has shown to have increase the abundance of *Akkermansia muciniphila* in gut of mice [40]. In addition it has been reported that increase in the abundance of *Akkermansia muciniphila* has negative correlation with occurrence of damage to mammary gland [40]. The distinct mucin degrading capacity of *Akkermansia muciniphila* may helps it in adapting to mammary gland and use it as alternate niche instead of its normal habitat i.e. intestine thus making this specie a potential candidate to be used as a probiotic [39]. Hence we suggest for its use in developing probiotics for use in the control of intrammamry infection in bovines.

Alpha diversity indices showed a higher diversity amongst healthy group samples than the other two groups which are in agreement with the findings of Hoque [41]. Clinical mastitis group was found to have lowest alpha diversity due to significant change in taxonomic profile of the quarter [42]. Beta diversity showed a clear separation of clinical mastitis sample as similar findings were earlier reported [41]. The difference in the taxonomic profile suggests that there is a variation in udder microbiota with the difference in health of the udder. The study has provided a base line data related to the specific breed i.e. Sahiwal but a deep insight is needed to explore the difference in milk microbiota in different management and husbandry practices.

## Conclusions

It is concluded that the diversity is lost in case of mastitis which is reflected at various levels and significant variation in milk microbiota composition between mastitic Sahiwal cattle and healthy Sahiwal cattle exists. The variation in abundance pattern of different taxa in groups may be used as a marker for identification of mastitis susceptibility. Microbial species which are abundant in healthy and subclinical mastitis groups but not detected from clinical mastitis may further be explored for their Probiotic potential for the prevention of mastitis in cattle especially further exploration of *Akkermansia muciniphila* specie is suggested.

### Supplementary Information

**Additional file 1: Supplementary table 1.** Farm Data from which samples were collected. **Supplementary table 2.** Animals Data: highlighted data shows samples processed for 16S r RNA gene base metagenomics.

**Additional file 2: Supplementary figure S1. **Heat-map plot of the relative abundance of different classes in Sahiwal cattle Milk Microbiota. **Supplementary figure S2.** Heat-map plot of the relative abundance of different orders in Sahiwal cattle Milk Microbiota.** Supplementary figure S3.** Heat-map plot of the relative abundance of different families in Sahiwal cattle Milk Microbiota. **Supplementary figure S4.** Heat-map plot of the relative abundance of different genra in Sahiwal cattle Milk Microbiota. **Supplementary figure S5.** Heat-map plot of the relative abundance of different species in Sahiwal cattle Milk Microbiota.

**Additional file 3: Supplementary table 3. **Represents legends for samples ID along with their status. These samples ID with their corresponding udder health status are used in all tables showing  percentage abundance of different taxa at individual samples level. **Supplementary table 4.** Sample wise percentage abundance of different abundant Phyla in milk microbiota of Sahiwal cattle. **Supplementary table 5.** Sample wise percentage abundance of different abundant Classes in milk microbiota of Sahiwal cattle. **Supplementary table 6.** Sample wise percentage abundance of different abundant Orders in milk microbiota of Sahiwal cattle. **Supplementary table 7.** Sample wise abundance of different abundant Families in milk microbiota of Sahiwal cattle. **Supplementary table 8.** Sample wise percentage abundance of different abundant Genra in milk microbiota of Sahiwal cattle. **Supplementary table 9.** Sample wise percentage abundance of different abundant species in milk microbiota of Sahiwal cattle. **Supplementary table 10.** Group wise percentage abundance of different abundant Classes in milk microbiota of Sahiwal cattle. **Supplementary table 11. **Group wise percentage abundance of different abundant Families in milk microbiota of Sahiwal cattle.

## Data Availability

The datasets generated and/or analyzed during the current study are available in the national center for biotechnology information (NCBI) under accessions number PRJNA950945 (https://www.ncbi.nlm.nih.gov/bioproject/?term=PRJNA950945).
