# Supplementary material for: Investigation of milk microbiota of healthy and mastitic Sahiwal cattle
Source: BMC Microbiol. 2023 Oct 24;23:304. doi: 10.1186/s12866-023-03051-0 (PMC10594912; doi:10.1186/s12866-023-03051-0)
Supplement: Supplementary file 1 — Additional file 1: Supplementary table 1. Farm Data from which samples were collected. Supplementary table 2. Animals Data: highlighted data shows samples processed for 16S r RNA gene base metagenomics. [file 12866_2023_3051_MOESM1_ESM.docx]

**Supplementary table.1: Farm Data from which samples were collected**

| **Farm**  **Reference**  **ID** | **address** | **Total cattle** | **male** | **female** | **less than 1 year** | **1-3 years** | **above 3 years** | **Feeding**  **Stall**  **Sometime grazing** | | | **Milking Practice** | **Floor type** | **Cleaning of animal** | **Milking parlor condition** |
| --- | --- | --- | --- | --- | --- | --- | --- | --- | --- | --- | --- | --- | --- | --- |
|  |  |  |  |  |  |  |  | **green fodder** | **commericial feed** | **Wheat Straw** |  |  |  |  |
| E | Okara | 20 | 1 | 19 | 4 | 3 | 13 | yes | seldom | Yes | Hand Milking | Concrete+kacha floor Mix | Monthly | Not very clean |
| F | Okara | 16 | 1 | 15 | 2 | 3 | 10 | Yes | seldom | Yes | Hand Milking | Concrete+kacha floor Mix | Monthly | Not very clean |
| G | Okara | 13 | 0 | 13 | 2 | 2 | 9 | Yes | seldom | Yes | Hand Milking | Concrete+kacha floor Mix | Monthly | Not very clean |
| H | Okara | 12 | 1 | 11 | 3 | 1 | 8 | Yes | seldom | Yes | Hand Milking | Concrete+kacha floor Mix | Monthly | Not very clean |

**Supplementary table.2: Animals Data: highlighted data shows samples processed for 16S r RNA gene base metagenomics**

| **Farm Reference ID** | **Sample ID** | **Metagenomic ID** | **Age** | **Parity** | **Lactation stage** | **Milk yield** | **Mastitis history** | **Antibiotic history** | **Date antibiotic given** | **Physical examination udder/milk** | **CMT Score** | **Somatic cell Count**  **(SCC)**  **X(1000)** | **Surf field test** | **Animal Status** |
| --- | --- | --- | --- | --- | --- | --- | --- | --- | --- | --- | --- | --- | --- | --- |
| E | M-39 |  | 4 | 1 | early | 8 | NIL | NIL | NIL | Normal | N | 153 | Negative | Healthy |
| E | M-40 |  | 6 | 2 | late | 5 | Yes | NIL | NIL | Abnormal | T | 379 | Positive | Clinical |
| E | M-41 | SM-41 | 5 | 2 | early | 5 | Yes | NIL | NIL | Abnormal | 2 | 532 | Positive | Clinical |
| E | M-42 | SM-42 | 5 | 2 | Mid | 4 | NIL | NIL | NIL | Abnormal | 1 | 401 | Positive | Clinical |
| E | M-43 | SM-43 | 4 | 1 | early | 5 | Yes | NIL | NIL | Abnormal | 1 | 397 | Positive | Clinical |
| E | M-44 | SM-44 | 6 | 3 | early | 5 | Yes | Nil | NIL | Abnormal | 2 | 455 | Positive | Clinical |
| E | M-45 | SM-45 | 5 | 2 | early | 6 | Nil | Nil | Nil | Abnormal | 2 | 469 | Positive | Clinical |
| E | M-46 |  | 4 | 2 | late | 5 | Yes | Nil | Nil | Abnormal | T | 345 | Positive | Clinical |
| E | M-47 |  | 6 | 2 | early | 7 | Nil | Nil | Nil | Normal | N | 133 | Negative | Healthy |
| E | M-48 |  | 5 | 2 | early | 7 | Nil | Nil | Nil | Normal | T | 247 | Positive | Sub Clinical |
| E | M-49 |  | 4 | 1 | late | 8 | Yes | Nil | NIL | Normal | T | 288 | Positive | Sub Clinical |
| E | M-50 |  | 5 | 2 | early | 9 | Yes | YES | NIL | Normal | T | 256 | Positive | Sub Clinical |
| F | M-51 | SS-51 | 6 | 2 | early | 8 | Nil | Nil | Nil | Normal | T | 245 | Positive | Sub Clinical |
| F | M-52 |  | 6 | 3 | Late | 9 | Nil | Nil | NIL | Normal | T | 266 | Positive | Sub Clinical |
| F | M-53 | SS-52 | 5 | 2 | early | 8 | Nil | Nil | NIL | Normal | T | 253 | Positive | Sub Clinical |
| F | M-54 |  | 6 | 3 | late | 8 | NIL | Nil | NIL | Normal | T | 277 | Positive | Sub Clinical |
| F | M-55 | SS-55 | 6 | 2 | early | 7 | Nil | Nil | Nil | Normal | T | 262 | Positive | Sub Clinical |
| F | M-56 | SS-56 | 5 | 2 | mid | 7 | Nil | Nil | NIL | Normal | T | 274 | Positive | Sub Clinical |
| F | M-57 |  | 6 | 2 | early | 7 | Nil | Nil | NIL | Abnormal | 1 | 393 | Positive | Clinical |
| F | M-58 |  | 5 | 2 | late | 6 | NIl | Nil | Nil | Abnormal | 1 | 380 | Positive | Clinical |
| G | M-59 |  | 4 | 2 | mid | 11 | NIL | NIL | Nil | Normal | N | 154 | Negative | Healthy |
| G | M-60 |  | 4 | 2 | mid | 10 | NIL | NIL | Nil | Normal | N | 65 | Negative | Healthy |
| G | M-61 |  | 6 | 3 | mid | 9 | Yes | Nil | Nil | Normal | N | 123 | Negative | Healthy |
| G | M-62 |  | 5 | 2 | late | 6 | Nil | NIl | NIL | Abnormal | 2 | 455 | Positive | Clinical |
| G | M-63 |  | 4 | 2 | early | 10 | Nil | Nil | Nil | Normal | N | 113 | Negative | Healthy |
| F | M-64 | SS-64 | 6 | 3 | early | 8 | Nil | Nil | Nil | Normal | T | 241 | Positive | Sub Clinical |
| F | M-65 |  | 5 | 2 | late | 9 | Nil | Nil | Nil | Normal | T | 219 | Positive | Sub Clinical |
| H | M-66 | SH-66 | 6 | 3 | early | 8 | Nil | Nil | Nil | Normal | N | 98 | Negative | Healthy |
| H | M-67 | SH-67 | 5 | 2 | early | 9 | Nil | Nil | Nil | Normal | N | 107 | Negative | Healthy |
| H | M-68 |  | 6 | 2 | early | 9 | Nil | Nil | NIL | Normal | T | 241 | Positive | Sub Clinical |
| G | M-69 |  | 4 | 2 | early | 10 | Nil | Nil | Nil | Normal | N | 108 | Negative | Healthy |
| G | M-70 |  | 6 | 3 | early | 7 | Nil | Nil | NIL | Abnormal | 2 | 441 | Positive | Clinical |
| H | M-71 | SH-71 | 6 | 3 | early | 9 | Nil | Nil | Nil | Normal | N | 67 | Negative | Healthy |
| H | M-72 | SH-72 | 5 | 2 | early | 10 | Nil | Nil | NIL | Normal | N | 83 | Negative | Healthy |
| H | M-73 |  | 4 | 2 | early | 6 | Nil | Nil | Nil | Abnormal | 2 | 468 | Positive | Clinical |
| H | M-74 | SH-74 | 6 | 2 | early | 10 | Nil | Nil | NIL | Normal | N | 137 | Negative | Healthy |

CMT(California Mastitis Test) score= N= Negative, T=trace, 1= weak positive, 2= and above = Distinct Positive

The Colored rows indicates the samples processed for 16S rRNA gene base metagenomic analysis

=Healthy, =Clinical Mastitis =Subclinical Mastitis,
