# Supplementary material for: Investigation of milk microbiota of healthy and mastitic Sahiwal cattle
Source: BMC Microbiol. 2023 Oct 24;23:304. doi: 10.1186/s12866-023-03051-0 (PMC10594912; doi:10.1186/s12866-023-03051-0)
Supplement: Supplementary file 2 — Additional file 2: Supplementary figure S1. Heat-map plot of the relative abundance of different classes in Sahiwal cattle Milk Microbiota. Supplementary figure S2. Heat-map plot of the relative abundance of different orders in Sahiwal cattle Milk Microbiota. Supplementary figure S3. Heat-map plot of the relative abundance of different families in Sahiwal cattle Milk Microbiota. Supplementary figure S4. Heat-map plot of the relative abundance of different genra in Sahiwal cattle Milk Microbiota. Supplementary figure S5. Heat-map plot of the relative abundance of different species in Sahiwal cattle Milk Microbiota. [file 12866_2023_3051_MOESM2_ESM.docx]

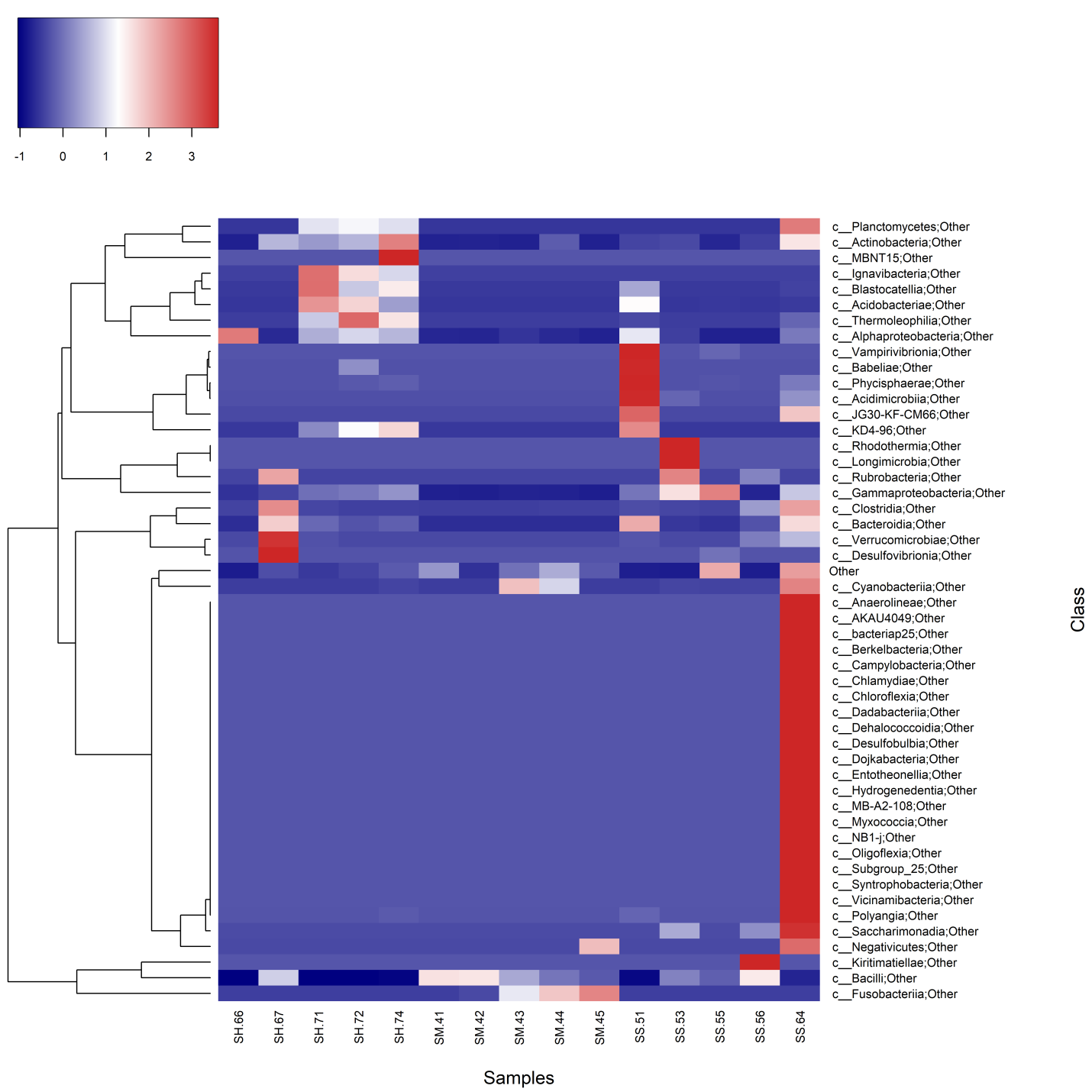


**Supplementary figure.S1**: **Heat-map plot of the relative abundance of different classes in Sahiwal cattle Milk Microbiota.** Healthy (n=5), Clinical Mastitis (n=5) and Subclinical Mastitis (n=5).Dendrograms show the clustering of different Classes in the same group


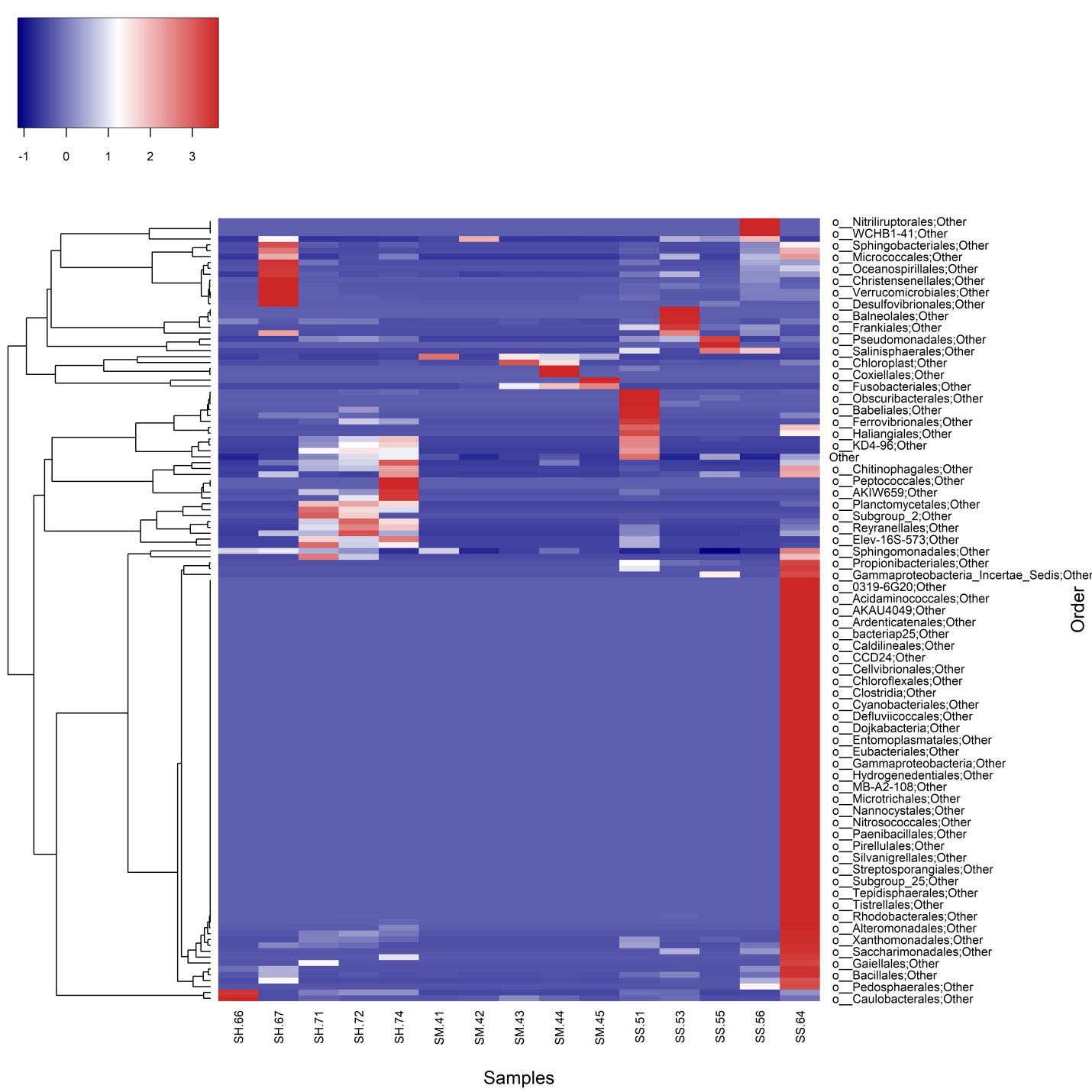


**Supplementary figure.S2**: **Heat-map plot of the relative abundance of different orders in Sahiwal cattle Milk Microbiota.** Healthy (n=5), Clinical Mastitis (n=5) and Subclinical Mastitis (n=5).Dendrograms show the clustering of different Orders in the same group


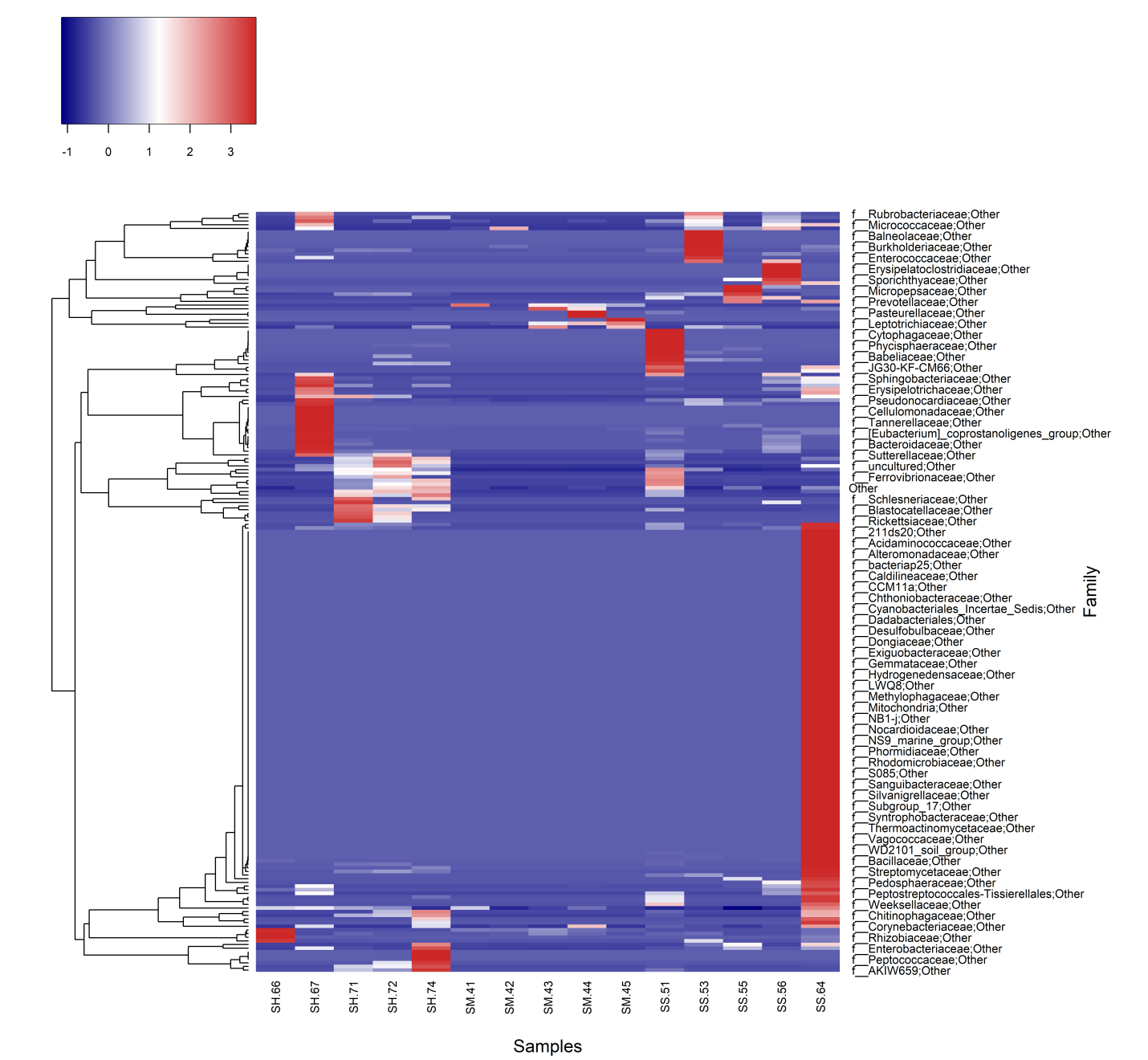


**Supplementary figure.S3**: **Heat-map plot of the relative abundance of different families in Sahiwal cattle Milk Microbiota.** Healthy (n=5), Clinical Mastitis (n=5) and Subclinical Mastitis (n=5).Dendrograms show the clustering of different families in the same group


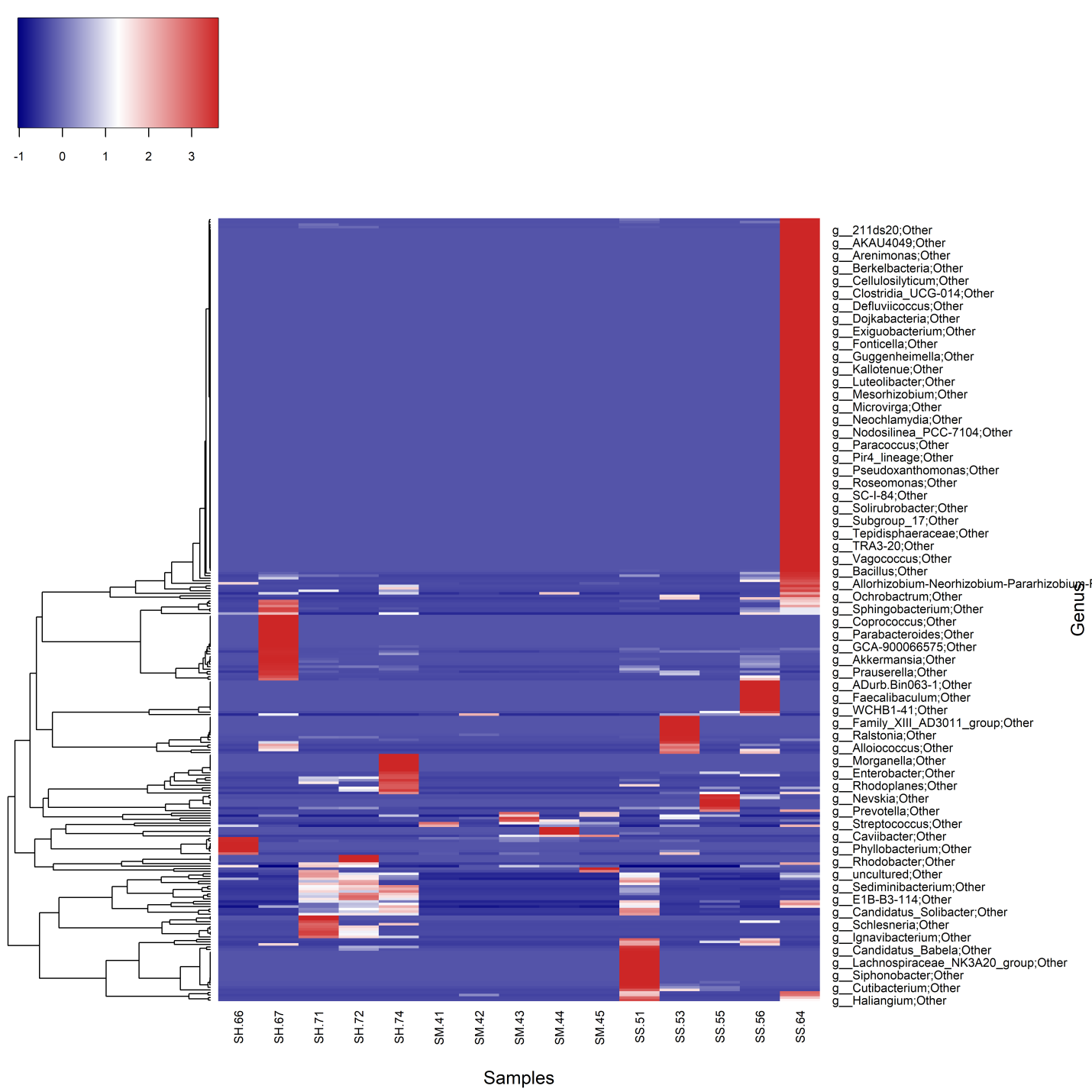
 **Supplementary figure.S4**: **Heat-map plot of the relative abundance of different genra in Sahiwal cattle Milk Microbiota.** Healthy (n=5), Clinical Mastitis (n=5) and Subclinical Mastitis (n=5).Dendrograms show the clustering of different genra in the same group


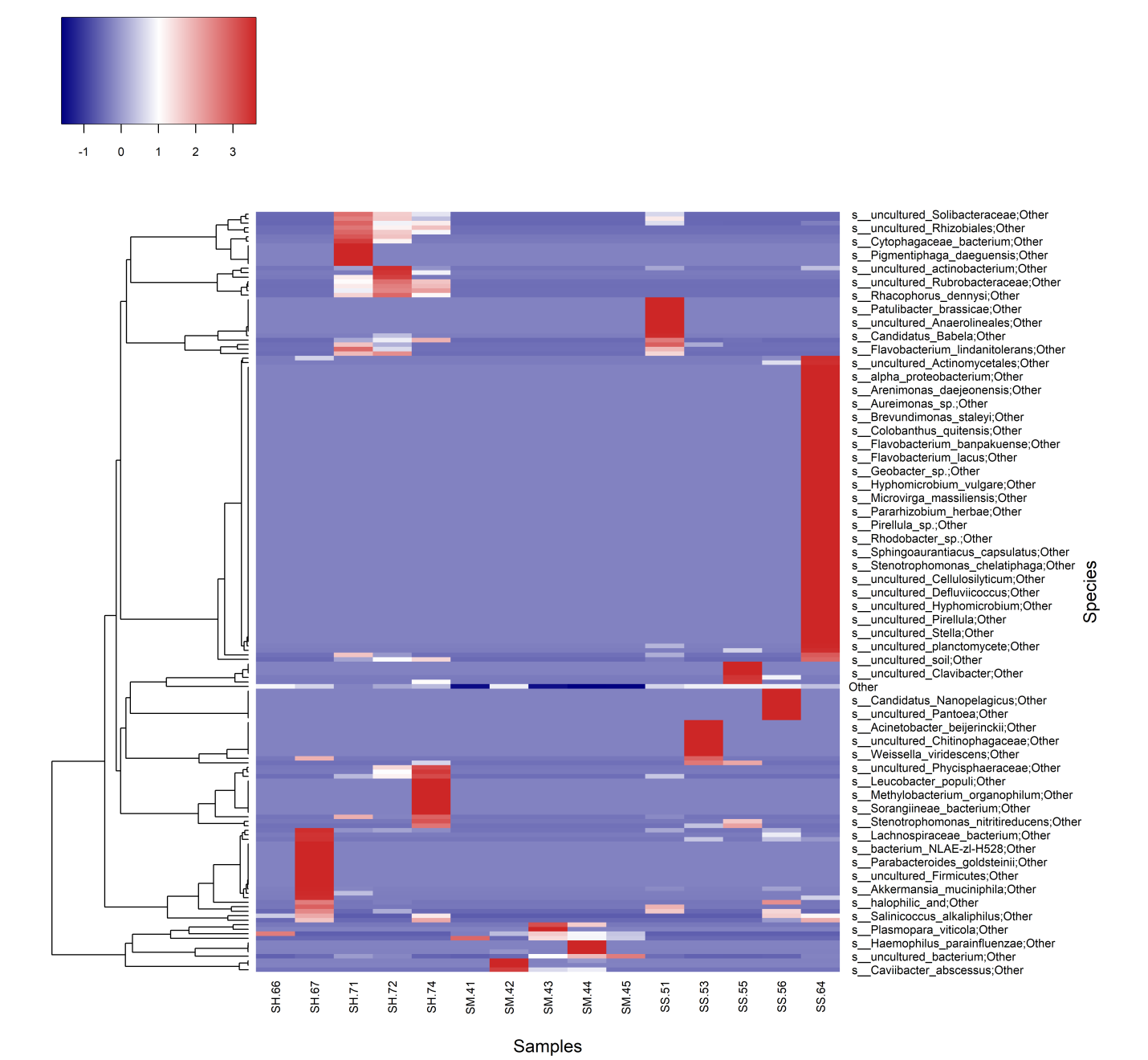


**Supplementary figure.S5**: **Heat-map plot of the relative abundance of different species in Sahiwal cattle Milk Microbiota.** Healthy (n=5), Clinical Mastitis (n=5) and Subclinical Mastitis (n=5).Dendrograms show the clustering of different species in the same group
