# Supplementary material for: Investigation of milk microbiota of healthy and mastitic Sahiwal cattle
Source: BMC Microbiol. 2023 Oct 24;23:304. doi: 10.1186/s12866-023-03051-0 (PMC10594912; doi:10.1186/s12866-023-03051-0)
Supplement: Supplementary file 3 — Additional file 3: Supplementary table 3. Represents legends for samples ID along with their status. These samples ID with their corresponding udder health status are used in all tables showing percentage abundance of different taxa at individual samples level. Supplementary table 4. Sample wise percentage abundance of different abundant Phyla in milk microbiota of Sahiwal cattle. Supplementary table 5. Sample wise percentage abundance of different abundant Classes in milk microbiota of Sahiwal cattle. Supplementary table 6. Sample wise percentage abundance of different abundant Orders in milk microbiota of Sahiwal cattle. Supplementary table 7. Sample wise abundance of different abundant Families in milk microbiota of Sahiwal cattle. Supplementary table 8. Sample wise percentage abundance of different abundant Genra in milk microbiota of Sahiwal cattle. Supplementary table 9. Sample wise percentage abundance of different abundant species in milk microbiota of Sahiwal cattle. Supplementary table 10. Group wise percentage abundance of different abundant Classes in milk microbiota of Sahiwal cattle. Supplementary table 11. Group wise percentage abundance of different abundant Families in milk microbiota of Sahiwal cattle. [file 12866_2023_3051_MOESM3_ESM.docx]

**Percentage abundance of different abundant taxa at different levels in milk microbiota of Sahiwal cattle (individual and group wise)**

**Supplementary table.3: Represents legends for samples ID along with their status .These samples ID with their corresponding udder health status are used in all tables showing percentage abundance of different taxa at individual samples level.**

| Sample ID | Status |
| --- | --- |
| SH-66 | Healthy |
| SH-67 | Healthy |
| SH-71 | Healthy |
| SH-72 | Healthy |
| SH-74 | Healthy |
| SM-41 | Clinical Mastitis |
| SM-42 | Clinical Mastitis |
| SM-43 | Clinical Mastitis |
| SM-44 | Clinical Mastitis |
| SM-45 | Clinical Mastitis |
| SS-51 | Sub clinical Mastitis |
| SS-53 | Sub clinical Mastitis |
| SS-55 | Sub clinical Mastitis |
| SS-56 | Sub clinical Mastitis |
| SS-64 | Sub clinical Mastitis |

**Supplementary table.4:Sample wise percentage abundance of different abundant Phyla in milk microbiota of Sahiwal cattle.**

| **phylum** | **SH-66** | **SH-67** | **SH-71** | **SH-72** | **SH-74** | **SM-41** | **SM-42** | **SM-43** | **SM-44** | **SM-45** | **SS-51** | **SS-53** | **SS-55** | **SS-56** | **SS-64** |
| --- | --- | --- | --- | --- | --- | --- | --- | --- | --- | --- | --- | --- | --- | --- | --- |
| *Proteobacteria* | 99.61 | 9.24 | 50.33 | 62.13 | 58.63 | 2 | 1.61 | 5.99 | 3.04 | 0.76 | 65.84 | 53.34 | 66.38 | 1.66 | 53.07 |
| *Firmicutes* | 0.39 | 76.36 | 0.27 | 0.43 | 1.88 | 97.9 | 96.03 | 56.9 | 39.78 | 30.6 | 3.37 | 44 | 33.03 | 95.78 | 18.73 |
| *Acidobacteriota* | 0 | 0 | 27.75 | 16.24 | 12.63 | 0 | 0 | 0 | 0 | 0 | 12.89 | 0 | 0.07 | 0 | 1.93 |
| *Actinobacteriota* | 0 | 6.87 | 7.1 | 11.28 | 19.46 | 0.1 | 0.15 | 0.05 | 2.66 | 0.08 | 3.21 | 2.11 | 0.27 | 1.49 | 12.22 |
| *Bacteroidota* | 0 | 4.08 | 13.86 | 8.91 | 6.19 | 0 | 0 | 0 | 0 | 0 | 4.67 | 0.28 | 0.06 | 0.56 | 3.91 |
| *Fusobacteriota* | 0 | 0 | 0 | 0 | 0 | 0 | 2.21 | 34.9 | 53.29 | 68.56 | 0.08 | 0 | 0 | 0 | 0 |

**Milk Sample type.SH= healthy, SM=Clinical Mastitis, SS=Subclinical Mastitis.**

**Supplementary table.5: Sample wise percentage abundance of different abundant Classes in milk microbiota of Sahiwal cattle.**

| **Class** | **SH-66** | **SH-67** | **SH-71** | **SH-72** | **SH-72** | **SM-41** | **SM-42** | **SM-43** | **SM-44** | **SM-45** | **SS-51** | **SS-53** | **SS-55** | **SS-56** | **SS-64** |
| --- | --- | --- | --- | --- | --- | --- | --- | --- | --- | --- | --- | --- | --- | --- | --- |
| *Alphaproteobacteria* | 95.98 | 2.23 | 36.13 | 46.32 | 38.13 | 1.49 | 1.26 | 5.04 | 2.25 | 0.62 | 50.54 | 7.97 | 0.36 | 0.39 | 23.03 |
| *Gammaproteobacteria* | 3.63 | 7.01 | 14.20 | 15.82 | 20.59 | 0.51 | 0.35 | 0.95 | 0.79 | 0.14 | 15.30 | 45.37 | 66.02 | 1.27 | 30.06 |
| *Bacilli* | 0.31 | 69.95 | 0.13 | 0.40 | 1.86 | 97.90 | 96.03 | 56.90 | 39.75 | 30.49 | 3.08 | 43.85 | 32.93 | 93.96 | 12.59 |
| *Clostridia* | 0.08 | 6.41 | 0.14 | 0.03 | 0.02 | 0.00 | 0.00 | 0.00 | 0.04 | 0.03 | 0.29 | 0.14 | 0.10 | 1.82 | 6.04 |
| *Acidobacteriae* | 0.00 | 0.00 | 14.21 | 10.97 | 4.50 | 0.00 | 0.00 | 0.00 | 0.00 | 0.00 | 8.86 | 0.00 | 0.07 | 0.00 | 0.14 |
| *Actinobacteria* | 0.00 | 6.66 | 5.34 | 6.61 | 16.51 | 0.10 | 0.15 | 0.05 | 2.66 | 0.08 | 1.62 | 1.81 | 0.27 | 1.45 | 11.37 |
| *Bacteroidia* | 0.00 | 4.08 | 0.90 | 0.58 | 0.76 | 0.00 | 0.00 | 0.00 | 0.00 | 0.00 | 4.67 | 0.15 | 0.06 | 0.56 | 3.91 |
| *Blastocatellia* | 0.00 | 0.00 | 13.54 | 5.26 | 8.13 | 0.00 | 0.00 | 0.00 | 0.00 | 0.00 | 4.03 | 0.00 | 0.00 | 0.00 | 0.36 |
| *Cyanobacteriia* | 0.00 | 0.00 | 0.00 | 0.00 | 0.05 | 0.00 | 0.00 | 2.16 | 1.23 | 0.00 | 0.00 | 0.06 | 0.04 | 0.06 | 2.77 |
| *Fusobacteriia* | 0.00 | 0.00 | 0.00 | 0.00 | 0.00 | 0.00 | 2.21 | 34.90 | 53.29 | 68.56 | 0.08 | 0.00 | 0.00 | 0.00 | 0.00 |
| *Ignavibacteria* | 0.00 | 0.00 | 12.97 | 8.34 | 5.44 | 0.00 | 0.00 | 0.00 | 0.00 | 0.00 | 0.00 | 0.00 | 0.00 | 0.00 | 0.00 |

**Milk Sample type.SH= healthy, SM=Clinical Mastitis, SS=Subclinical Mastitis.**

**Supplementary table.6: Sample wise percentage abundance of different abundant Orders in milk microbiota of Sahiwal cattle.**

| **Order** | **SH-66** | **SH-67** | **SH-71** | **SH-72** | **SH-74** | **SM-41** | **SM-42** | **SM-43** | **SM-44** | **SM-45** | **SS-51** | **SS-53** | **SS-55** | **SS-56** | **SS-64** |
| --- | --- | --- | --- | --- | --- | --- | --- | --- | --- | --- | --- | --- | --- | --- | --- |
| *Rhizobiales* | 65.11 | 1.48 | 8.55 | 11.98 | 11.99 | 0.24 | 0.08 | 0.34 | 0.06 | 0.15 | 6.83 | 5.49 | 0.19 | 0.05 | 11.76 |
| *Caulobacterales* | 30.19 | 0.00 | 1.56 | 0.77 | 0.73 | 0.57 | 1.00 | 4.44 | 1.79 | 0.21 | 1.49 | 2.20 | 0.00 | 0.15 | 2.49 |
| *Burkholderiales* | 3.63 | 0.84 | 3.02 | 2.91 | 0.14 | 0.32 | 0.33 | 0.94 | 0.25 | 0.07 | 1.68 | 27.25 | 1.22 | 0.29 | 2.75 |
| *Staphylococcales* | 0.17 | 63.83 | 0.00 | 0.04 | 0.55 | 5.68 | 95.18 | 0.40 | 0.14 | 0.51 | 2.36 | 40.42 | 32.07 | 92.56 | 2.83 |
| *Corynebacteriales* | 0.00 | 2.36 | 5.11 | 6.41 | 16.41 | 0.00 | 0.13 | 0.00 | 2.61 | 0.00 | 0.78 | 0.23 | 0.03 | 0.22 | 5.79 |
| *Enterobacterales* | 0.00 | 5.34 | 0.50 | 0.21 | 12.90 | 0.11 | 0.03 | 0.02 | 0.11 | 0.07 | 0.68 | 0.92 | 3.84 | 0.73 | 11.80 |
| *Flavobacteriales* | 0.00 | 0.26 | 0.19 | 0.13 | 0.00 | 0.00 | 0.00 | 0.00 | 0.00 | 0.00 | 0.42 | 0.00 | 0.00 | 0.18 | 2.02 |
| *Fusobacteriales* | 0.00 | 0.00 | 0.00 | 0.00 | 0.00 | 0.00 | 2.21 | 34.90 | 53.29 | 68.56 | 0.09 | 0.00 | 0.00 | 0.00 | 0.00 |
| *Ignavibacteriales* | 0.00 | 0.00 | 13.15 | 8.56 | 5.59 | 0.00 | 0.00 | 0.00 | 0.00 | 0.00 | 0.00 | 0.00 | 0.00 | 0.00 | 0.00 |
| *Parvibaculales* | 0.00 | 0.00 | 23.95 | 28.82 | 23.19 | 0.00 | 0.00 | 0.00 | 0.00 | 0.00 | 41.48 | 0.00 | 0.04 | 0.00 | 0.00 |
| *Pseudomonadales* | 0.00 | 0.26 | 10.30 | 12.50 | 7.50 | 0.08 | 0.00 | 0.00 | 0.00 | 0.00 | 12.92 | 17.13 | 60.52 | 0.00 | 7.66 |

**Milk Sample type.SH= healthy, SM=Clinical Mastitis, SS=Subclinical Mastitis.**

**Supplementary table.7: Sample wise abundance of different abundant Families in milk microbiota of Sahiwal cattle.**

| **Family** | **SH-66** | **SH-67** | **SH-71** | **SH-72** | **SH-74** | **SM-41** | **SM-42** | **SM-43** | **SM-44** | **SM-45** | **SS-51** | **SS-53** | **SS-55** | **SS-56** | **SS-64** |
| --- | --- | --- | --- | --- | --- | --- | --- | --- | --- | --- | --- | --- | --- | --- | --- |
| *Rhizobiaceae* | 51.49 | 0.53 | 0.00 | 0.00 | 0.00 | 0.05 | 0.02 | 0.21 | 0.00 | 0.15 | 0.07 | 0.09 | 0.00 | 0.00 | 4.52 |
| *Caulobacteraceae* | 30.19 | 0.00 | 1.57 | 0.80 | 0.77 | 0.57 | 1.00 | 4.44 | 1.79 | 0.21 | 1.50 | 2.20 | 0.00 | 0.15 | 2.42 |
| *Staphylococcaceae* | 0.17 | 63.98 | 0.00 | 0.04 | 0.58 | 5.68 | 95.18 | 0.40 | 0.14 | 0.51 | 2.38 | 40.42 | 32.07 | 92.56 | 2.85 |
| *Corynebacteriaceae* | 0.00 | 1.64 | 0.00 | 0.00 | 1.77 | 0.00 | 0.13 | 0.00 | 2.61 | 0.00 | 0.43 | 0.23 | 0.03 | 0.22 | 3.13 |
| *Enterobacteriaceae* | 0.00 | 5.35 | 0.50 | 0.22 | 13.12 | 0.11 | 0.03 | 0.02 | 0.11 | 0.07 | 0.59 | 0.92 | 3.84 | 0.73 | 3.13 |
| *Ignavibacteriaceae* | 0.00 | 0.00 | 13.27 | 8.88 | 5.89 | 0.00 | 0.00 | 0.00 | 0.00 | 0.00 | 0.00 | 0.00 | 0.00 | 0.00 | 0.00 |
| *Leptotrichiaceae* | 0.00 | 0.00 | 0.00 | 0.00 | 0.00 | 0.00 | 2.21 | 34.90 | 53.29 | 68.56 | 0.00 | 0.00 | 0.00 | 0.00 | 0.00 |
| *Parvibaculaceae* | 0.00 | 0.00 | 24.18 | 29.90 | 24.41 | 0.00 | 0.00 | 0.00 | 0.00 | 0.00 | 41.81 | 0.00 | 0.04 | 0.00 | 0.00 |
| *Pseudomonadaceae* | 0.00 | 0.26 | 10.38 | 12.97 | 2.58 | 0.05 | 0.00 | 0.00 | 0.00 | 0.00 | 13.02 | 16.40 | 57.53 | 0.00 | 4.09 |
| *Streptococcaceae* | 0.00 | 0.00 | 0.00 | 0.00 | 0.00 | 92.14 | 0.85 | 53.40 | 39.45 | 27.43 | 0.05 | 0.00 | 0.00 | 0.00 | 0.00 |

**Milk Sample type.SH= healthy, SM=Clinical Mastitis, SS=Subclinical Mastitis.**

**Supplementary table.8: Sample wise percentage abundance of different abundant Genra in milk microbiota of Sahiwal cattle.**

| **Genus** | **SH-66** | **SH-67** | **SH-71** | **SH-72** | **SH-74** | **SM-41** | **SM-42** | **SM-43** | **SM-44** | **SM-45** | **SS-51** | **SS-53** | **SS-55** | **SS-56** | **SS-64** |
| --- | --- | --- | --- | --- | --- | --- | --- | --- | --- | --- | --- | --- | --- | --- | --- |
| *Novosphingobium* | 0.34 | 0.00 | 0.39 | 0.33 | 0.05 | 0.03 | 0.10 | 0.24 | 0.16 | 0.11 | 0.00 | 0.00 | 0.00 | 0.20 | 0.12 |
| *Caviibacter* | 0.00 | 0.00 | 0.00 | 0.00 | 0.00 | 0.00 | 2.21 | 34.91 | 53.32 | 68.56 | 0.00 | 0.00 | 0.00 | 0.00 | 0.00 |
| *Corynebacterium* | 0.00 | 1.75 | 0.00 | 0.00 | 1.89 | 0.00 | 0.13 | 0.00 | 2.61 | 0.00 | 0.19 | 0.09 | 0.00 | 0.23 | 3.44 |
| *Escherichia-Shigella* | 0.00 | 4.88 | 0.19 | 0.22 | 0.49 | 0.11 | 0.03 | 0.02 | 0.11 | 0.07 | 0.35 | 0.64 | 0.71 | 0.42 | 0.33 |
| *Ignavibacterium* | 0.00 | 0.00 | 13.48 | 9.01 | 6.31 | 0.00 | 0.00 | 0.00 | 0.00 | 0.00 | 0.00 | 0.00 | 0.00 | 0.00 | 0.00 |
| *Lactobacillus* | 0.00 | 0.63 | 0.00 | 0.00 | 1.09 | 0.00 | 0.00 | 1.60 | 0.09 | 1.55 | 0.05 | 1.27 | 0.89 | 0.00 | 0.00 |
| *Parvibaculum* | 0.00 | 0.00 | 24.55 | 30.34 | 26.18 | 0.00 | 0.00 | 0.00 | 0.00 | 0.00 | 43.08 | 0.00 | 0.00 | 0.00 | 0.00 |
| *Pseudomonas* | 0.00 | 0.27 | 10.53 | 13.16 | 2.76 | 0.05 | 0.00 | 0.00 | 0.00 | 0.00 | 13.42 | 16.68 | 58.22 | 0.00 | 4.49 |
| *Staphylococcus* | 0.00 | 66.86 | 0.00 | 0.04 | 0.26 | 5.68 | 95.24 | 0.40 | 0.14 | 0.51 | 2.45 | 41.01 | 32.46 | 93.04 | 2.84 |
| *Streptococcus* | 0.00 | 0.00 | 0.00 | 0.00 | 0.00 | 92.22 | 0.85 | 53.42 | 39.48 | 27.43 | 0.00 | 0.00 | 0.00 | 0.00 | 0.00 |
| *Uncultured* | 0.00 | 1.22 | 15.57 | 8.87 | 10.39 | 0.00 | 0.00 | 0.00 | 0.00 | 0.00 | 10.06 | 0.13 | 0.09 | 0.27 | 5.90 |

**Milk Sample type.SH= healthy, SM=Clinical Mastitis, SS=Subclinical Mastitis.**

**Supplementary table.9:.Sample wise percentage abundance of different abundant species in milk microbiota of Sahiwal cattle.**

|  | | | |  |  |  |  |  |  |  |  |  |  |  |  |
| --- | --- | --- | --- | --- | --- | --- | --- | --- | --- | --- | --- | --- | --- | --- | --- |
| **Species** | **SH-66** | **SH-67** | **SH-71** | **SH-72** | **SH-74** | **SM-41** | **SM-42** | **SM-43** | **SM-44** | **SM-45** | **SS-51** | **SS-53** | **SS-55** | **SS-56** | **SS-64** |
| *Novosphingobium capsulatum* | 52.11 | 0.00 | 0.00 | 0.00 | 0.00 | 0.04 | 3.05 | 0.26 | 0.17 | 0.11 | 0.00 | 0.00 | 0.00 | 0.00 | 0.00 |
| *Akkermansia muciniphila* | 0.00 | 17.74 | 0.09 | 0.00 | 0.00 | 0.00 | 0.00 | 0.00 | 0.00 | 0.00 | 0.59 | 0.00 | 0.00 | 7.58 | 0.00 |
| *Corynebacterium bovis* | 0.00 | 0.00 | 0.00 | 0.00 | 0.00 | 0.00 | 4.02 | 0.00 | 2.64 | 0.00 | 0.00 | 0.00 | 0.00 | 0.00 | 0.00 |
| *Ignavibacterium album* | 0.00 | 0.00 | 28.64 | 26.87 | 23.07 | 0.00 | 0.00 | 0.00 | 0.00 | 0.00 | 0.00 | 0.00 | 0.00 | 0.00 | 0.00 |
| *Lactobacillus fermentum* | 0.00 | 0.00 | 0.00 | 0.00 | 0.58 | 0.00 | 0.00 | 0.00 | 0.00 | 0.00 | 0.00 | 14.01 | 14.53 | 0.00 | 0.00 |
| *Pseudomonas sp.* | 0.00 | 0.00 | 0.54 | 0.25 | 0.00 | 0.00 | 0.00 | 0.00 | 0.00 | 0.00 | 2.82 | 2.38 | 0.00 | 0.00 | 0.00 |
| *Streptococcus dysgalactiae* | 0.00 | 0.00 | 0.00 | 0.00 | 0.00 | 99.96 | 17.77 | 58.88 | 40.61 | 28.55 | 0.00 | 0.00 | 0.00 | 0.00 | 0.00 |
| *uncultured Acidobacteriales* | 0.00 | 0.00 | 1.34 | 4.13 | 9.82 | 0.00 | 0.00 | 0.00 | 0.00 | 0.00 | 21.79 | 0.00 | 2.94 | 0.00 | 0.00 |
| *uncultured actinobacterium* | 0.00 | 0.00 | 0.20 | 2.71 | 0.27 | 0.00 | 0.00 | 0.00 | 0.00 | 0.00 | 0.80 | 0.00 | 0.00 | 0.00 | 0.78 |
| *uncultured Phyllobacteriaceae* | 0.00 | 0.00 | 0.30 | 0.98 | 1.16 | 0.00 | 0.00 | 0.00 | 0.00 | 0.00 | 0.00 | 0.00 | 0.00 | 0.00 | 0.00 |
| *uncultured Rhizobiales* | 0.00 | 0.00 | 0.17 | 0.13 | 0.23 | 0.00 | 0.00 | 0.00 | 0.00 | 0.00 | 0.00 | 0.00 | 0.00 | 0.00 | 0.00 |
| *uncultured Rubrobacteraceae* | 0.00 | 0.00 | 1.77 | 5.50 | 4.90 | 0.00 | 0.00 | 0.00 | 0.00 | 0.00 | 0.00 | 0.00 | 0.00 | 0.00 | 0.00 |

**Milk Sample type.SH= healthy, SM=Clinical Mastitis, SS=Subclinical Mastitis.**

**Supplementary table.10:Group wise percentage abundance of different abundant Classes in milk microbiota of Sahiwal cattle.**

| **Class** | **Healthy** |  | **Clinical Mastitis** |  | **Subclinical Mastitis** |  |
| --- | --- | --- | --- | --- | --- | --- |
|  | **Mean** | **SEM** | **Mean** | **SEM** | **Mean** | **SEM** |
| *Alphaproteobacteria* | 43.76 | 15.08 | 2.13 | 0.77 | 16.46 | 9.47 |
| *Gammaproteobacteria* | 12.25 | 3.06 | 0.55 | 0.15 | 31.60 | 11.32 |
| *Bacilli* | 14.53 | 13.86 | 64.21 | 14.03 | 37.28 | 15.90 |
| *Clostridia* | 1.34 | 1.27 | 0.01 | 0.01 | 1.68 | 1.14 |
| *Acidobacteriae* | 5.94 | 2.88 | 0.00 | 0.00 | 1.81 | 1.76 |
| *Actinobacteria* | 7.02 | 2.67 | 0.61 | 0.51 | 3.30 | 2.03 |
| *Bacteroidia* | 1.26 | 0.72 | 0.00 | 0.00 | 1.87 | 1.00 |
| *Fusobacteriia* | 0.00 | 0.00 | 31.79 | 13.62 | 0.02 | 0.02 |

**SEM=Standard error mean**

**Supplementary table.11:Group wise percentage abundance of different abundant Families in milk microbiota of Sahiwal cattle.**

| **Family** | **Healthy** |  | **Clinical Mastitis** |  | **Subclinical Mastitis** |  |
| --- | --- | --- | --- | --- | --- | --- |
|  | **Mean** | **SEM** | **Mean** | **SEM** | **Mean** | **SEM** |
| *Rhizobiaceae* | 10.40 | 10.27 | 0.08 | 0.04 | 0.93 | 0.90 |
| *Caulobacteraceae* | 6.67 | 5.89 | 1.60 | 0.76 | 1.25 | 0.51 |
| *Staphylococcaceae* | 12.95 | 12.76 | 20.38 | 18.73 | 34.06 | 16.50 |
| *Corynebacteriaceae* | 0.68 | 0.42 | 0.55 | 0.52 | 0.81 | 0.58 |
| *Enterobacteriaceae* | 3.84 | 2.52 | 0.07 | 0.02 | 1.84 | 0.68 |
| *Ignavibacteriaceae* | 5.61 | 2.57 | 0.00 | 0.00 | 0.00 | 0.00 |
| *Leptotrichiaceae* | 0.00 | 0.00 | 31.79 | 13.62 | 0.00 | 0.00 |
| *Parvibaculaceae* | 15.70 | 6.49 | 0.00 | 0.00 | 8.37 | 8.36 |
| *Pseudomonadaceae* | 5.24 | 2.70 | 0.01 | 0.01 | 18.21 | 10.26 |
| *Streptococcaceae* | 0.00 | 0.00 | 42.66 | 15.09 | 0.01 | 0.01 |

**SEM=Standard error mean**
